# FASN inhibits ferroptosis in breast cancer via USP5 palmitoylation-dependent regulation of GPX4 deubiquitination

**DOI:** 10.1186/s13046-025-03548-8

**Published:** 2025-10-14

**Authors:** Zhiwen Qian, Ying Jiang, Yun Cai, Erli Gao, Cenzhu Wang, Jianfeng Dong, Fengxu Wang, Lu Liu, Danping Wu, Feng Zhang, Yida Wang, Xin Ning, Qi Li, Yilan You, Yanfang Gu, Jie Mei, Xinyuan Zhao, Yan Zhang

**Affiliations:** 1 Department of Oncology, Wuxi Maternal and Child Health Hospital, Nanjing Medical University, Wuxi, Jiangsu China; 2 Department of Oncology, School of Medicine, Women’s Hospital of Jiangnan University, Jiangnan University, WuxiWuxi, Jiangsu China; 3 Central Laboratory, Jintan Affiliated Hospital of Jiangsu University, Changzhou, Jiangsu People’s Republic of China; 4 Department of General Surgery, The First Affiliated Hospital of Soochow University, Suzhou, China; 5 Department of Oncology, The Affiliated Wuxi People’s Hospital of Nanjing Medical University, Wuxi People’s Hospital, Wuxi Medical Center, Nanjing Medical University, Wuxi, China; 6 Department of Pathology, Affiliated Women’s Hospital of Jiangnan University, Wuxi, Jiangsu, China; 7 School of Public Health, Nantong University, Nantong, China; 8 The First Clinical Medicine College, Nanjing Medical University, Nanjing, Jiangsu China

**Keywords:** FASN, Ferroptosis, Breast cancer, USP5, Palmitoylation

## Abstract

Increasing studies have reported that dysregulated lipid metabolism is an independent risk factor for breast cancer (BC); it would be, therefore, enlightening to investigate the relationship between metabolic reprogramming and the tumor microenvironment in the future. Ferroptosis, a novel form of programmed cell death, is characterized by glutathione (GSH) depletion and inactivation of glutathione peroxidase 4 (GPX4), the central regulator of the antioxidant system. While the close association between fatty acid metabolism and ferroptosis has been studied in various diseases, the interplay between the key fatty acid metabolic enzyme fatty acid synthase (FASN) and ferroptosis in BC remains unexplored. At the beginning of the current study, we demonstrated that FASN expression positively correlates with an immune-cold tumor microenvironment in BC. Subsequent findings revealed that FASN knockdown promotes GPX4 degradation-induced ferroptosis, thereby enhancing the efficacy of anti-programmed cell death protein 1 (PD-1) immunotherapy. Co-immunoprecipitation coupled with mass spectrometry (IP/MS) and co-IP experiments demonstrated that ubiquitin specific protease 5 (USP5) stabilizes GPX4 by binding to and deubiquitinating it. Furthermore, knockdown of FASN inhibited the palmitoylation of USP5, reducing its interaction with GPX4 and consequently increasing GPX4 ubiquitination and degradation. Our results demonstrate that FASN suppresses ferroptosis in BC by stabilizing GPX4 via USP5-mediated mechanisms, highlighting FASN inhibition as a potential therapeutic approach to enhance immunotherapy response.

**Supplementary Information:**

The online version contains supplementary material available at 10.1186/s13046-025-03548-8.

Breast cancer (BC) is the most prevalent malignant neoplasm among women across the globe [1]. Despite advancements in detection and treatment, BC remains a formidable adversary, continuing to be a major cause of morbidity and mortality [2, 3]. Currently, while surgical removal during the early stages continues to be a primary treatment for BC [4], the therapeutic landscape has evolved to encompass a broader array of personalized approaches, employing a complex series of therapeutic strategies [5]. In the realm of immunotherapy, the presence of tumor-infiltrating lymphocytes (TILs) is like a beacon of hope, indicating a pre-existing immune response against the tumor, which correlates with a positive response to chemotherapy and an improved prognosis [6, 7]. However, the battle is far from over. Metabolic reprogramming, a particularly intriguing hallmark of BC, has emerged as a new front in this war. In recent years, there has been significant research into the intricate mechanisms of metabolic reprogramming in BC, leading to the utilization of genes associated with these processes as targets for clinical therapy [8].

In 2012, Dixon et al. coined the term 'ferroptosis' to describe a novel, nonapoptotic, and iron-dependent form of cell death [9]. This type of cell death is characterized by the excessive accumulation of lipid peroxidation, which occurs as a consequence of metabolic dysfunction [10]. Since its discovery, ferroptosis has been hailed as a potential 'silver bullet' in BC treatment. Numerous studies have reported its important role, not only as a 'killer' itself but also as an enhancer of chemotherapy and immunotherapy [11–13]. However, despite these promising findings, the molecular mechanisms of ferroptosis remain shrouded in mystery, with many regulatory factors and signaling pathways still unclear.

Fatty acid synthase (FASN) is a key enzyme in lipid metabolism, catalyzing the conversion of acetyl-CoA and malonyl-CoA into long-chain fatty acids, including palmitic and stearic acids. High expression levels of FASN have been identified as a biomarker for BC [14]. FASN not only facilitates the epithelial-mesenchymal transition (EMT) and migration in BC cells, potentially driving tumor invasion and metastasis, but also significantly contributes to angiogenesis [15, 16]. In recent years, an increasing number of studies have revealed a close relationship between FASN's direct product and a particular type of protein post-translational modification known as palmitoylation [17–19]. S-palmitoylation is a reversible protein modification process in which palmitic acid covalently binds to the thiol group of cysteine residues in protein molecules [20], thereby exerting a significant influence on the structure and function of proteins. This modification regulates protein stability, function, and intracellular localization, affecting a wide range of biological processes in cells under both physiological and pathological conditions [21, 22].

In this study, we used immunoprecipitation-mass spectrometry (IP-MS) to screen for and evaluate particular proteins that interact with glutathione peroxidase 4(GPX4) in order to clarify the main processes by which FASN stabilizes GPX4 protein production. We found that ubiquitin specific protease 5(USP5) is a significant binding protein of GPX4. Interestingly, USP5's binding to GPX4 was diminished when FASN expression was suppressed, although USP5 expression in the total protein remained the same. Given FASN's essential role in both lipid metabolism and the palmitoylation modification that controls substrate protein levels and functions, we investigated its potential involvement in regulating USP5 binding to GPX4. Here, we demonstrate that FASN enhances the S-palmitoylation of USP5, which in turn promotes the protein stability of GPX4. Notably, combined treatment with the FASN inhibitor orlistat and anti-programmed cell death protein 1 (PD-1) therapy synergistically enhanced ferroptosis induction and significantly suppressed tumor growth. Our findings indicate that FASN-mediated S-palmitoylation of USP5 is a crucial mechanism for stabilizing GPX4 to inhibit ferroptosis, and propose a potential therapeutic strategy.

## Results

### Expression distribution and clinical significance of FASN in BC

**Fig. 1:**
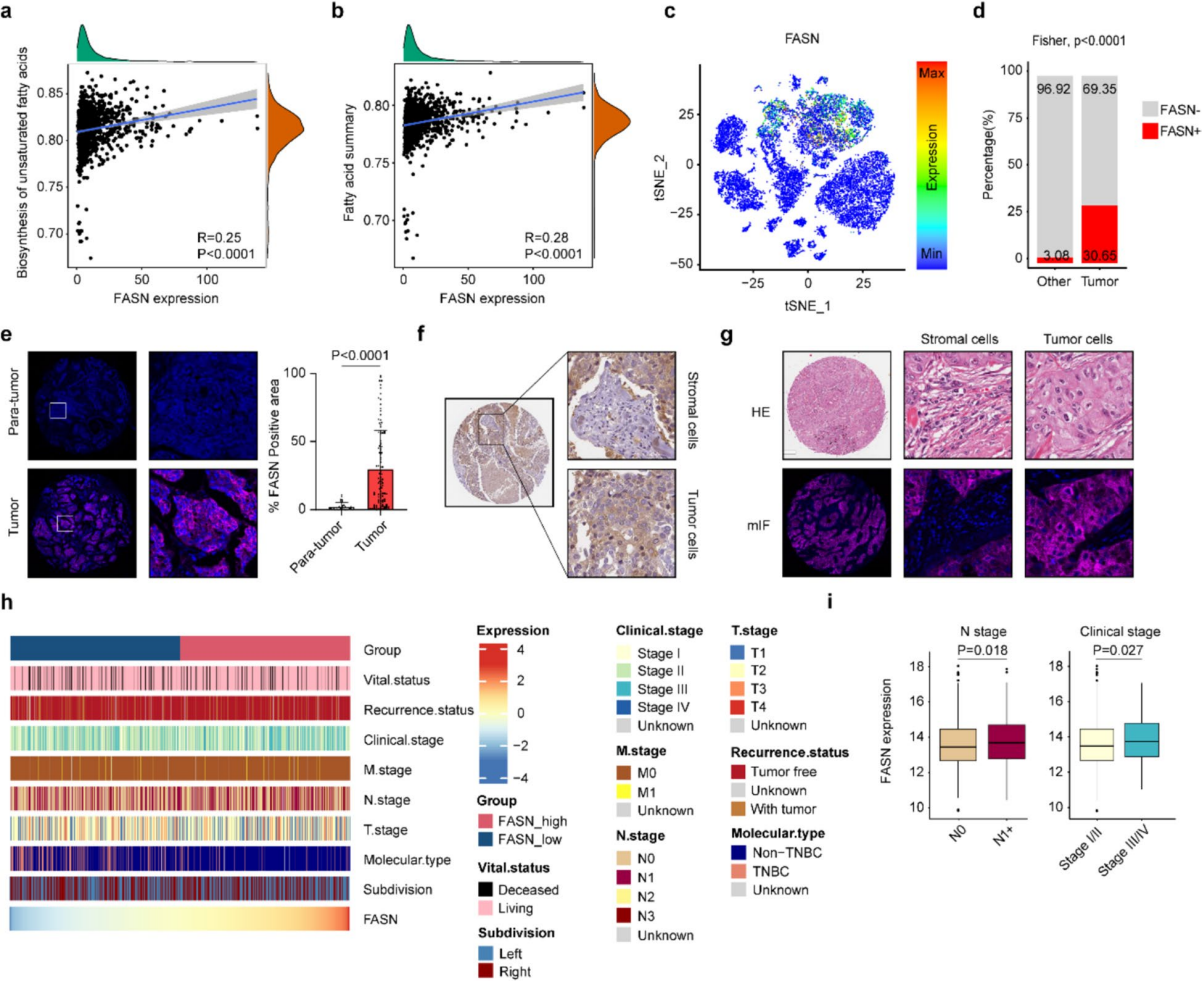
FASN expression patterns and metabolic associations in breast cancer. **a**, **b** Association between FASN and fatty acid metabolism pathways including biosynthesis of unsaturated fatty acids (**a**) and fatty acid summary (**b**). **c** scRNA-seq data (*n* = 19,600 cells from 5 patients) analysis of FASN expression distribution across cellular populations. **d** Binary classification of cells based on FASN expression (> 0). Bar plots compare FASN + proportions between tumor cells and non-tumor cells. **e** Multiplex immunofluorescence (mIF) of FASN (magenta) with DAPI (blue). Right: Quantification of FASN + positive area fraction. **f**, **g** Representative immunohistochemistry (IHC) and mIF staining of FASN in tumor cells and stromal cells. H&E staining (**g**, up) serves as spatial reference for mIF analysis. **h**, **i** Association between FASN expression and clinicopathological characteristics in TCGA-BRCA cohort. Scale bars: 50 μm (**e**- **g**). Groups were compared by Fisher's exact test (**d**), two-tailed unpaired t-test (**e**, right) or Wilcoxon rank-sum test (**i**). P < 0.05 was considered to be statistically significant. IHC images of FASN in tumor and stromal cells were obtained from the Human Protein Atlas (HPA) database (https://www.proteinatlas.org)

Emerging evidence indicates that aberrant fatty acid metabolism contributes significantly to oncogenic transformation across multiple cancer types, particularly in BC. The enzymatic regulators governing fatty acid biosynthesis and β-oxidation pathways are now recognized as pivotal mediators driving BC cell proliferation, metastatic dissemination, and invasive potential [23, 24]. As a pivotal enzyme in fatty acid metabolism, FASN is closely associated with key metabolic pathways including fatty acid biosynthesis, fatty acid elongation, fatty acid degradation, and unsaturated fatty acid biosynthesis (Fig. 1a, b, Supplementary Fig. 1a-c). In the last few decades, high expression of FASN has been found to be associated with poor prognosis in some cancers, such as cervical, breast, and gastric cancers. Bioinformatic analysis via the GEPIA2 (http://gepia2.cancer-pku.cn/) online tool demonstrated that FASN expression was significantly upregulated in BC tissues relative to normal breast tissues (Supplementary Fig. 1d). To elucidate the expression distribution of FASN in various cells within BC tissues, we collected single-cell RNA sequencing (scRNA-seq) data (GSE180286 [25]) from the primary tumor tissues of five BC patients who had not undergone any prior treatment. Using the classic cell-type-specific molecular markers reported in the literature, we preliminarily annotated these cells into seven major cell types (Supplementary Fig. 1e-i). Moreover, we identified cell-type-specific highly expressed genes to validate the cell annotation results (Supplementary Fig. 1j, Supplementary Fig. 2a). Observation revealed that cells with high FASN expression tended to cluster within tumor cell populations (Fig. 1c). After that, we classified these data into two categories based on FASN expression: cells with FASN expression greater than zero were defined as FASN + cells, while the remaining cells were classified as FASN − cells. We then compared the proportions of FASN + and FASN − cells among tumor cells and non-tumor cells. The results showed that 30.65% of tumor cells were FASN + , which was significantly higher than the proportion of FASN + cells among non-tumor cells (3.08%) (Fig. 1d). To confirm the high expression level in BC, we applied multiplex immunofluorescence (mIF) to compare FASN protein levels between para-tumor and tumor (Fig. 1e). In addition, immunohistochemical (IHC) results from the HPA database (https://www.proteinatlas.org**)**, along with validation by HE staining and mIF, confirmed that FASN was highly expressed in tumor cells rather than in stromal cells within BC tissues (Fig. 1f, g).

To further investigate the relationship between FASN and clinical characteristics of BC (Fig. 1h, i), we downloaded the RNA sequencing (RNA-seq) data and corresponding clinical information of the BRCA dataset from The Cancer Genome Atlas (TCGA) database via the UCSC Xena website (https://xena.ucsc.edu/public). A total of 1,069 BC patient data with complete survival information were retained. Although FASN levels did not significantly differ among patients with different sampling sites or TM stages, it was significantly upregulated in patients with BC who had lymph node metastasis or a high clinical pathological stage (stage III/IV) (Supplementary Fig. 2b). This suggests that FASN overexpression is closely associated with pathological progression in BC. Collectively, these findings demonstrate that FASN expression correlates with clinical stage and lymph node metastasis in BC.

### FASN links to immunosuppressive tumor microenvironment in BC

Based on the spatial distribution of cytotoxic immune cells in the tumor microenvironment (TME), researchers classified tumors into three basic immune phenotypes: immune-excluded, immune-suppressed (cold), and immune-inflamed (hot) tumors [26]. The microenvironmental differences between hot and cold tumors significantly influence the effectiveness of immunotherapy. Hot tumors are characterized by high T cell infiltration, increased interferon-γ (IFN-γ) signaling, elevated programmed cell death-ligand 1(PD-L1) expression, and high tumor mutation burden (TMB), which makes them more susceptible to immune checkpoint inhibitors (ICIs) [27]. In contrast, cold tumors exhibit low T cell infiltration, low mutation burden, low MHC I expression, low PD-L1 expression, and the presence of immunosuppressive cell populations [28, 29]. The microenvironmental differences between hot and cold tumors largely determine the efficacy of immunotherapy.

**Fig. 2:**
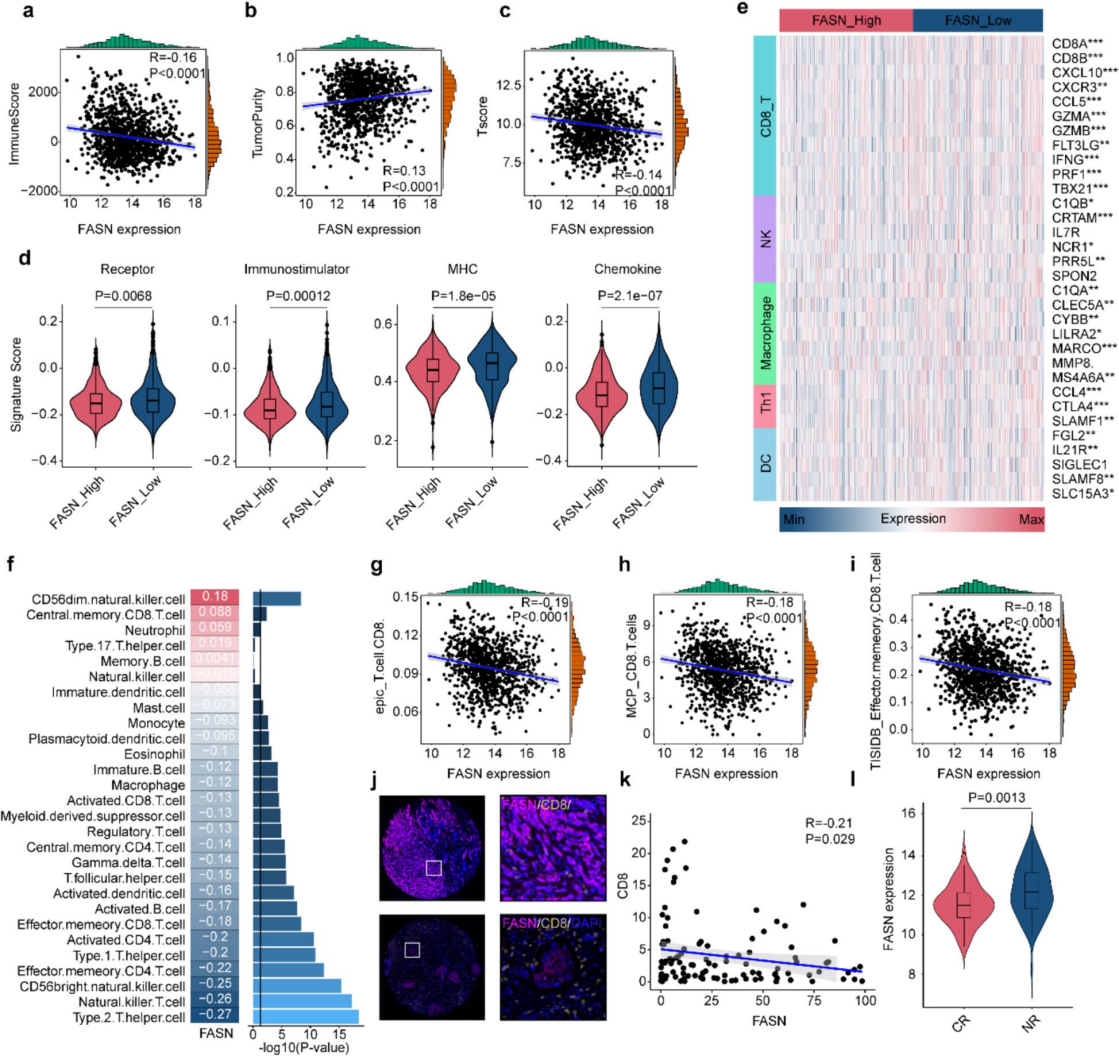
FASN correlates with tumor immunity in breast cancer. **a**-**c** ESTIMATE algorithm-derived tumor microenvironment characteristics in TCGA-BRCA: tumor purity (**a**), immune score (**b**), and T-cell inflammation score (**c**). **d** Differential expression of immunomodulatory genes between FASN-high and FASN-low groups (median cutoff). **e** Heatmap of immune cell signature gene expression in FASN-high vs. FASN-low groups (median cutoff). **f** Correlation between FASN expression and immune cell abundances. **g**-**i** Scatter plots showing correlations between FASN expression and CD8^+^ T-cell abundance predicted by EPIC (**g**), MCP-counter (**h**), and TISIDB (**i**). **j**,** k** Representative multiplex immunofluorescence (mIF) images demonstrating expression of FASN and CD8 in tumor cells (**j**) with Pearson correlation analysis (**k**). **l** FASN expression in immunotherapy-treated cohort (CR: complete response; NR: no response). Scale bars: 50 μm (**j**). Groups were compared by Wilcoxon rank-sum test (**d**,** l**). *P* < 0.05 was considered to be statistically significant

Previous studies have found that FASN is associated with immune suppression [30]; we subsequently explored the correlation between FASN and the status of the tumor immune microenvironment. We used the ESTIMATE algorithm to evaluate the tumor purity and immune score of each sample in the TCGA-BRCA cohort. We found that FASN expression levels were negatively correlated with the immune score and positively correlated with tumor purity (Fig. 2a, b). Moreover, FASN expression values were significantly negatively correlated with T-cell inflammation scores (Fig. 2c). These findings suggest that FASN may play a significant role in immune infiltration in BCs.

Based on the immune regulatory markers published in previous studies, we calculated the enrichment scores and found that the high FASN group exhibited significant downregulation of chemokines, receptors, MHC molecules, and immune regulators (Fig. 2d). Moreover, compared with the low FASN group, the high FASN group also showed downregulation of several common immune cell markers, such as CD8A and CD8B for CD8^+^ T cells, and C1QA and MMP8 for macrophages (Fig. 2e). Subsequently, according to the infiltration abundance of TIIC and the score of immune activation pathway, we found that the transcription level of FASN was negatively correlated with the infiltration abundance of most immune cells and the activation of immune pathway, especially CD8^+^ T cells (Fig. 2f, Supplementary Fig. 2c). In the high FASN group, the activity related to most steps of the cancer immune cycle was significantly decreased (Supplementary Fig. 2d). As expected, the high FASN group exhibited downregulation of most immune checkpoints, including LAG3 and IDO1 (Supplementary Fig. 2e). Besides, FASN was negatively correlated with CD8^+^ T cell infiltration (Fig. 2g-i). mIF and IHC staining further supported our findings (Fig. 2j, k, Supplementary Fig. 2f, g).

Additionally, we collected the gene expression profiles of BC immunotherapy cohorts (GSE194040 [31], with 95 cases, and GSE173839 [32], with 72 cases). We found that the immunotherapy-resistant group had higher levels of FASN expression (Fig. 2l). Moreover, compared with the low-FASN group, a higher proportion of immunotherapy-resistant BC patients were observed in the high-FASN group (Supplementary Fig. 2 h).

To sum up, this study found that FASN was highly correlated with tumor immune "cold" microenvironment, which may have diagnostic value in identifying the immunogenicity of BC.

### FASN promotes BC growth and metastasis in vitro

**Fig. 3:**
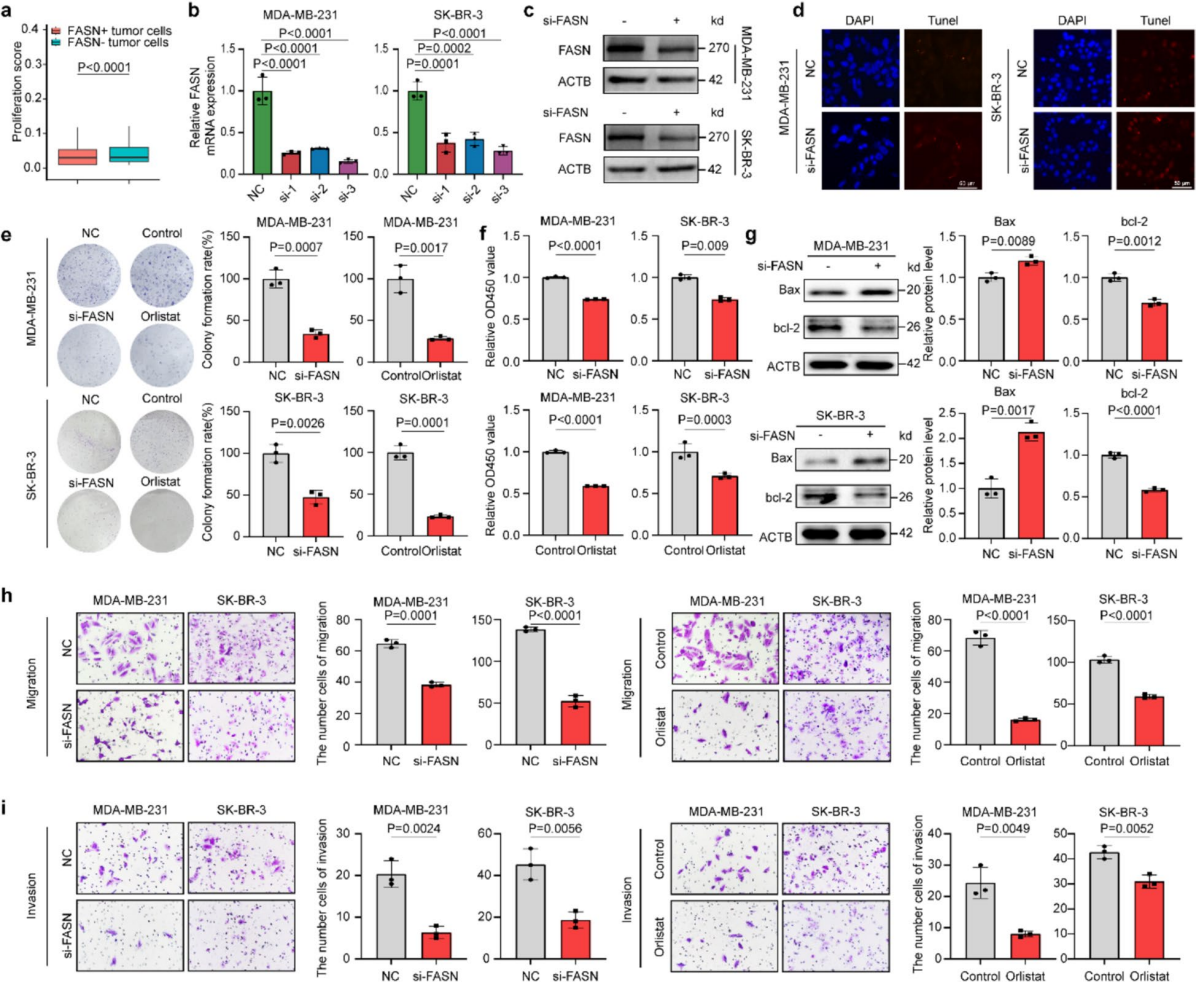
FASN modulates breast cancer malignancy by regulating tumor cell proliferation. **a** Proliferation score analysis of FASN + and FASN − tumor cells in scRNA-seq data (*n* = 19,600 cells from 5 patients). Proliferation scores were computed by applying the PercentageFeatureSet function in the Seurat package to quantify cell cycle-related gene expression signatures (see Methods). **b, c** Validation of FASN knockdown efficiency. qRT-PCR analysis of FASN mRNA levels in siRNA-transfected cells (normalized to GAPDH) (**b**) and western blot analysis of FASN protein expression (β-actin served as loading control) (**c**). **d** Apoptosis detection by TUNEL assay in FASN-knockdown cells (48 h post-transfection). Nuclei were stained with DAPI (blue); TUNEL-positive cells (red). **e, f** Functional consequences of FASN knockdown. **e**, Clonogenic survival assay (10-day culture). **f**, Cell viability measured by CCK-8 assay (48 h post-transfection). **g** Western blot analysis of apoptosis-related proteins (Bax and bcl-2) in FASN-knockdown MDA-MB-231 and SK-BR-3 cells. Quantification of protein levels (normalized to β-actin) is shown below the blots. **h, i** Effects of FASN knockdown on migration (**h**) and invasion (**i**). Representative images (left) and quantifications (right) of Transwell migration (top) and invasion (bottom) assays. Scale bars: 50 μm (**h**, **i**). Experiments were performed three times independently (**b**–**i**). Data were presented as mean ± SD (**b**–**f**). Groups were compared by two-tailed unpaired t-test (**f**, right of **e**, **g**–**i**) or one-way ANOVA followed by Fisher’s LSD test (**b**). *P* < 0.05 was considered to be statistically significant

In our previous study, we observed high expression of FASN in BC and FASN + tumor cells, which significantly activated G2-M checkpoint, glycolysis, cholesterol metabolism, and oxidative phosphorylation (Supplementary Fig. 3a-c). Considering FASN + tumor cells showed a higher level of proliferation (Fig. 3a), we knocked down FASN using siRNA in two cell lines, MDA-MB-231 and SK-BR-3, to elucidate its biological role in these BC cells. The efficiency of FASN knockdown was confirmed by qPCR and western blot (WB) analysis (Fig. 3b, c). The proliferative activity was assessed by CCK-8 and colony formation assays, and the migratory and invasive activities were assessed by transwell migration and invasion assays, respectively. As shown in Fig. 3f, a significant reduction in cell proliferation was observed in the si-FASN group (knockdown group) compared to the control group. Furthermore, the number of cell colonies was significantly increased in both MDA-MB-231 and SK-BR-3 cells with FASN knockdown (Fig. 3e). Simultaneously, we used TUNEL staining and WB to detect the expression levels of apoptosis-related proteins Bax and Bcl-2, in order to evaluate the effect of FASN knockdown on cell apoptosis. The results showed that compared with the control group, the TUNEL signal was enhanced in the si-FASN knockdown group (Fig. 3d); meanwhile, the expression of Bax was upregulated while the expression of Bcl-2 was downregulated (Fig. 3g). Additionally, transwell migration and invasion assays revealed that fewer BC cells migrated in the si-FASN group than in the si-Ctrl group (Fig. 3h, i). Then, we further observed the effects of orlistat, an inhibitor of FASN, on BC cell proliferation, migration, and invasion. We found that inhibiting FASN with orlistat led to similar decreases in these cellular activities (Fig. 3e, f, h, i). Different from FASN knockdown-induced apoptosis, orlistat treatment did not see a significant intracellular TUNEL signal enhancement effect, and apoptosis-related proteins Bax and Bcl-2 did not change significantly, and MDA-MB-231 and SKBR3 cells obtained this result (Supplementary Fig. 3d, e). Taken together, our findings suggest that FASN might be a favorable factor for the proliferation, migration, and invasion of BC cells. These results strongly support the role of FASN in promoting the progression of BC, prompting us to further investigate its potential mechanisms.

### FASN acts as an inhibitor of ferroptosis in BC cells

**Fig. 4:**
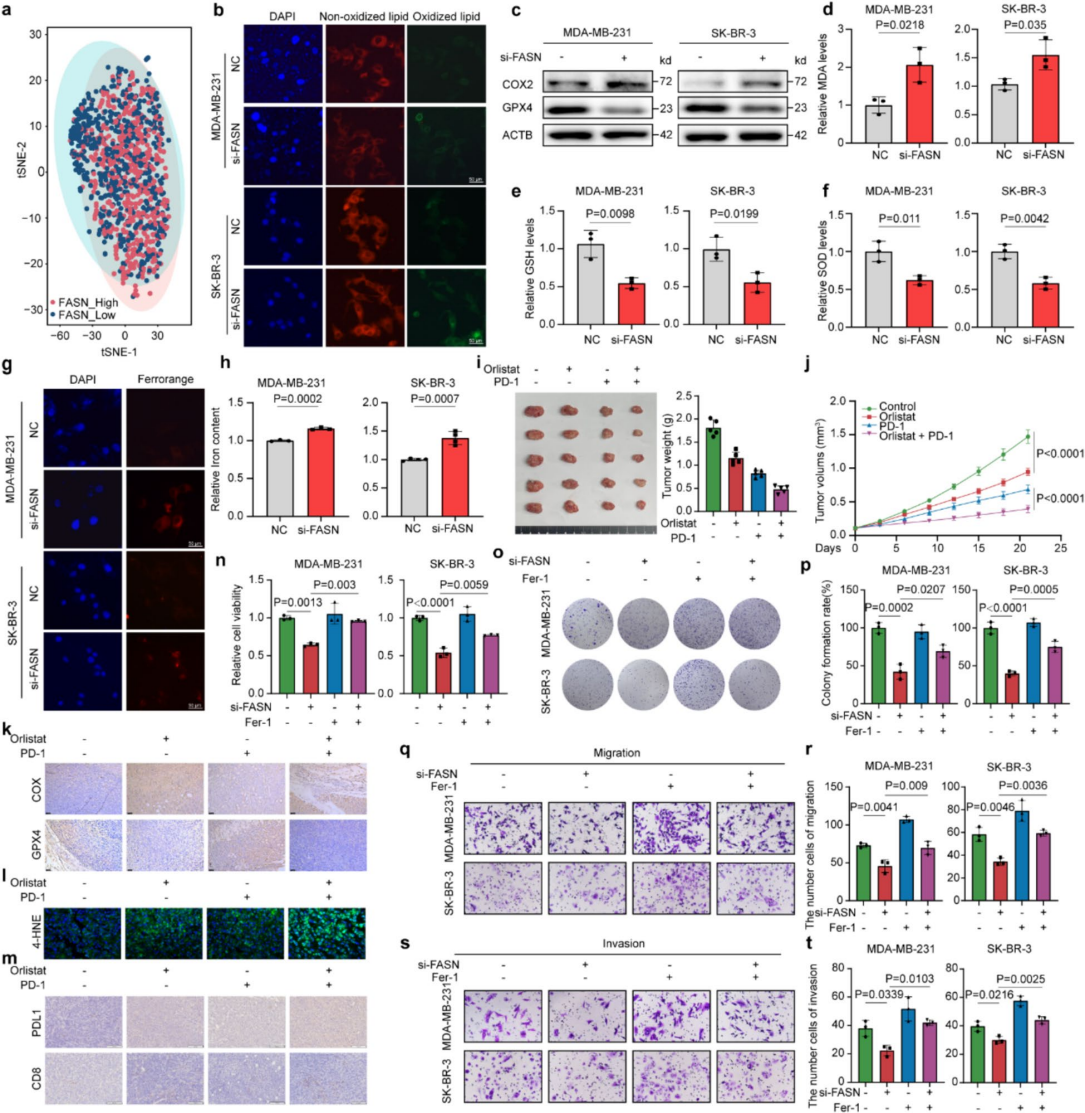
FASN modulates ferroptosis and enhances anti-PD-1 efficacy in breast cancer. **a** Transcriptomic profiling of breast cancer tumors from the TCGA-BRCA cohort (*n* = 1,069 patients). Patients were stratified into FASN-high and FASN-low groups based on median FASN expression. t-SNE analysis revealed ferroptosis-related transcriptional patterns between groups. **b** Lipid peroxidation detection in FASN-knockdown MDA-MB-231 and SK-BR-3 cells using C11-BODIPY 581/591 probe. Representative fluorescence images show oxidized lipid (green) and non-oxidized lipid (red). **c** Western blot analysis of GPX4 and COX-2 expression in MDA-MB-231 and SK-BR-3 cells following FASN knockdown. **d-f** Impact of FASN knockdown on oxidative stress and ferroptosis in MDA-MB-231 and SK-BR-3 cells. Cells were treated as in (**b**), then relative MDA (**d**), GSH (**e**), and SOD (**f**) content were detected by their corresponding kits. **g, h** FASN knockdown elevates intracellular ferrous iron (Fe^2^⁺) levels in MDA-MB-231 and SK-BR-3 cells, as quantified by FerroOrange fluorescence (**g**, 5 μM probe) and ferrozine assay (**h**). **i-m** Therapeutic efficacy of orlistat combined with anti-PD-L1 in BALB/c mice bearing 4T1 mammary tumors. Representative tumor images (**i**, left, *n* = 5) and comparative tumor weight analysis (**i**, right) across treatment groups. Tumor growth curves monitored over time (**j**). Immunohistochemical (IHC) staining of GPX4 and COX-2 in tumor tissues (**k**). 4-HNE levels detected by immunofluorescence (**l**, green). IHC analysis of PD-L1 and CD8 expression (**m**). **n-t** Effects of FASN knockdown combined with ferrostatin-1 (Fer-1, 5 μM) on malignant behaviors of breast cancer cells, including cell viability (**n**), clonogenic survival (**o**,** p**), migration (**q**,** r**) and invasion (**s**,** t**). Scale bars: 50 μm (**b**, **g**, **k-m**,** q**, **s**). Experiments were performed three times independently (**b**–**t**). Data were presented as mean ± SD (**d**–**f**, **h, i**,** n**, **p**, **r**, **t**). Groups were compared by two-tailed unpaired t-test (**d**–**f**,** h**) or one-way ANOVA followed by Fisher’s LSD test (**i**,** n**, **p**, **r**, **t**). *P* < 0.05 was considered to be statistically significant

FASN is considered to play a role in the inhibition of ferroptosis in various cancers. Following its activation, the balance between polyunsaturated fatty acids (PUFAs) and saturated fatty acids (SFAs) is disrupted [33]. As mentioned before, FASN has an oncogenic role in BC. Further analyses in the TCGA database, like t-distributed stochastic neighbor embedding (t-SNE) analysis, showed that there were obvious differences in ferroptosis transcription between high and low FASN groups (Fig. 4a, Supplementary Fig. 4a) and the overexpression of FASN was related to the inhibition of ferroptosis (Supplementary Fig. 4b, c).

To further elucidate the role of FASN in ferroptosis, we established FASN-knockdown BC cell models. Our results demonstrated that FASN knockdown significantly induced lipid peroxidation, which was confirmed by staining MDA-MB-231 and SK-BR-3 cells with the lipid peroxidation probe C11-BODIPY581/591 (Fig. 4b). GPX4, a key regulator of ferroptosis, exhibited the most significant decrease in FASN-knockdown cells, while COX2 levels were significantly increased. (Fig. 4c). This finding is consistent with the positive correlation between FASN and GPX4 observed in mIF staining (Supplementary Fig. 4 m, n). Additionally, we examined the changes in expression following orlistat treatment and found that expression levels were markedly altered (Supplementary Fig. 4d, e).

Ferroptosis is a unique form of regulated cell death, characterized by the excessive accumulation of lipid peroxides in the plasma membrane [34, 35]. Malondialdehyde (MDA) is the principal and most studied product of polyunsaturated fatty acid peroxidation, while Glutathione (GSH) consumption is closely related to lipid peroxidation reaction [36, 37]. Therefore, we next verified whether there were changes in the levels of MDA and GSH in FASN-knockdown cells and in cells treated with Orlistat. We observed that GSH levels decreased, while MDA levels increased (Fig. 4d, e). Lipid peroxidation is often accompanied by an increase in cellular oxidative stress [38]. Abnormalities in the cellular antioxidant defense system can be detected by measuring the activity of Superoxide Dismutase (SOD) [39]. In our study, the levels of SOD declined (Fig. 4f), indicating that FASN knockdown promotes the sensitivity of BC cells to ferroptosis. Lastly, we determined that the levels of cellular free iron were significantly higher in si-FASN cells than in control cells (Fig. 4g, h). Additionally, our study demonstrated that pharmacological inhibition of FASN using orlistat resulted in the same phenomenon (Supplementary Fig. 4f-j). Erastin effectively induces ferroptosis by targeting the core pathway of system Xc– – glutathione – GPX4. We found that knocking down FASN enhances the inducing effect of erastin (Supplementary Fig. 4 k, l). Thus, FASN knockdown contributed to the sensitization of tumor cells to ferroptosis in vitro.

### Ferrostatin-1 inhibits ferroptosis induced by FASN knockdown

As depicted in Figs. 3 and 4, we demonstrated that knockdown of FASN significantly induced a reduction in cell viability and the onset of ferroptosis. Consequently, we proceeded to evaluate the impact of the ferroptosis inhibitor Ferrostatin-1 (Fer-1) on the cytotoxicity associated with FASN knockdown. The diminished cell viability observed upon FASN knockdown was rescued by Fer-1 treatment, as evidenced in Fig. 4n-p. Furthermore, transwell migration and invasion assays disclosed that Fer-1 mitigated the inhibitory effects of FASN knockdown on cellular migration and invasion, as illustrated in Figs. 4q-t. Cells treated with orlistat showed similar results (Supplementary Fig. 5a-g). Taken together, these results confirmed that FASN acts as an inhibitor of ferroptosis in BC cells.

### FASN inhibition sensitizes tumors to immunotherapy via ferroptosis in vivo

Ferroptosis can limit the function of immunosuppressive cells, such as tumor-associated macrophages (TAMs) [40] and regulatory T cells (Tregs) [41], in immunologically cold tumors [42], transforming an immunosuppressive TME into an inflammatory TME that is rich in antitumor immune cells and enhancing the efficacy of ICIs [43]. Our previous findings confirmed that FASN ablation remodels the immune landscape by augmenting CD8^+^ T cell infiltration [30]. To further explore the role of FASN in regulating ferroptosis and cancer therapy in vivo, we evaluated the therapeutic efficacy of combining the FASN inhibitor (orlistat) with ICIs (anti-PD-1), which are known for their ability to block the binding of PD-1 to PD-L1 [44]. Our results demonstrated that orlistat combined with anti-PD-1 dramatically suppressed the subcutaneous 4T1 tumor growth and increased tumoral 4-HNE levels compared to single-agent treatment (Fig. 4i, j, l). Moreover, we measured expression of GPX4 and COX-2 in mice using IHC. Orlistat treatment significantly decreased GPX4 levels while markedly increasing COX-2 expression. (Fig. 4k). Notably, through analyzing the biomarkers of immune, we found that orlistat in combination with anti-PD-1 treatment increased the tumor-infiltrating CD8^+^ T cells and the expression of PD-L1, the biomarkers of immune (Fig. 4m, Supplementary Fig. 4t). This finding indicates that the combination therapy might remodel the tumor microenvironment to attract CD8^+^ T cells, thereby potentially enhancing the efficacy of T cell–mediated immunotherapy.

In order to evaluate whether orlistat and PD-1 inhibitor have obvious toxicity to mice, we investigated the blood lipid status and body weight. Interestingly, results showed that there is no difference between the treatment group and the control group (Supplementary Fig. 4o-s), which indicates the safety of orlistat. These findings suggest that orlistat treatment enhances ferroptosis in BC, leading to a significant increase in tumor sensitivity to immunotherapy.

### FASN stabilizes GPX4 by promoting USP5 binding to GPX4 and inhibiting its ubiquitination and degradation

**Fig. 5:**
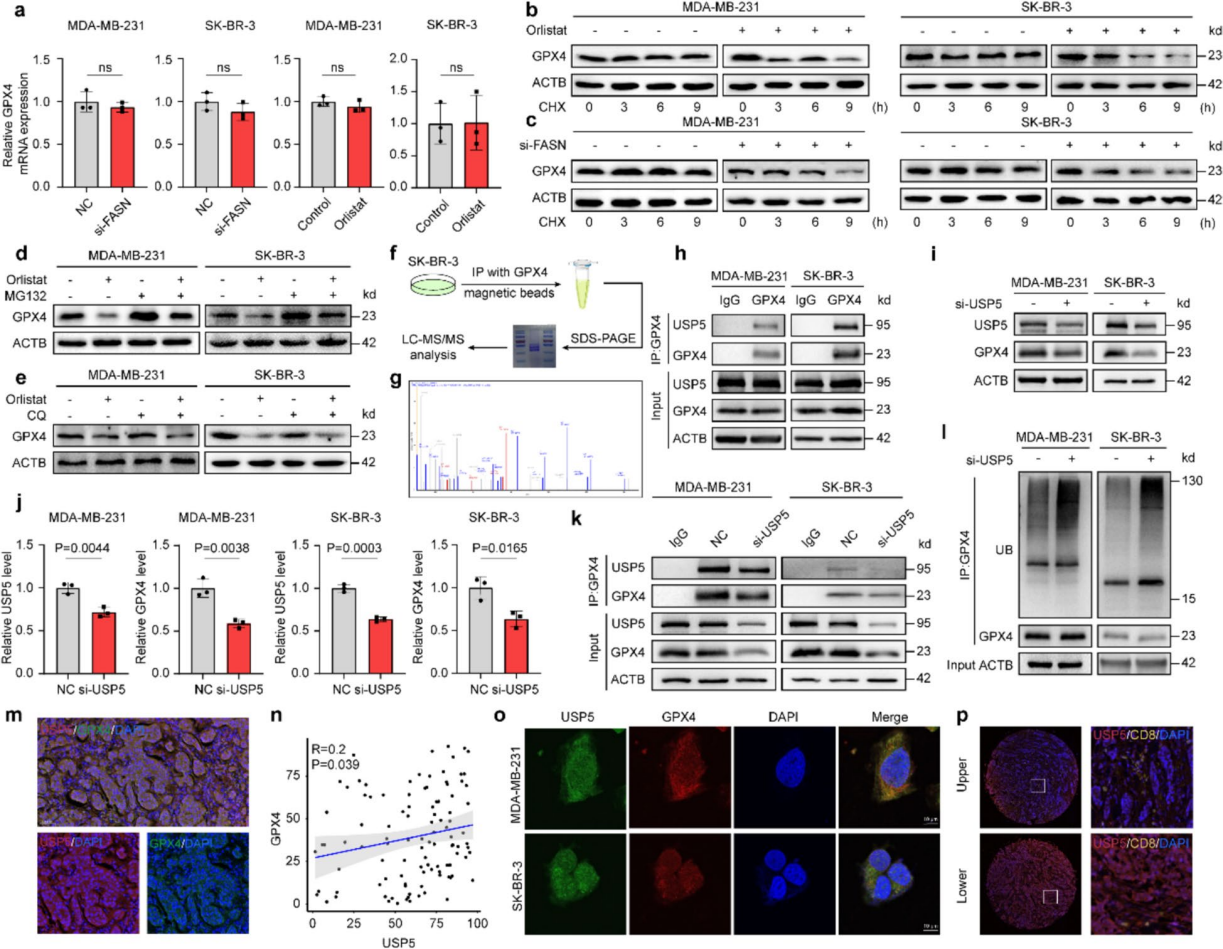
FASN knockdown inhibits USP5-mediated GPX4 deubiquitination. **a** qRT-PCR analysis of GPX4 mRNA in FASN-knockdown MDA-MB-231 and SK-BR-3 cells (normalized to GAPDH). **b**, **c** WB and quantification of GPX4 protein degradation in CHX-treated cells (10 μM, indicated times). **d**, **e** WB analysis of GPX4 in orlistat-treated cells with MG132 (**d**, 10 μM, 6 h) or chloroquine (**e**, CQ, 10 μM, 6 h). **f**, **g** Schematic of GPX4 interactor screening by IP-MS (**f**) and candidate protein list (**g**). **h** Co-IP of USP5 with anti-GPX4 antibody in breast cancer cells (IgG control). **i**, **j** WB and quantification of GPX4 in USP5-knockdown cells. **k** IP-WB analysis in siUSP5-expressing cells. **l** Ubiquitination assays in MG132-treated (10 μM, 6 h) cells transfected with siUSP5. **m, n** Multiplex immunofluorescence (mIF) images demonstrating expression of USP5 and GPX4 in tumor cells (**m**) with Pearson correlation analysis (**n**). **o** Endogenous GPX4 (red) and USP5 (green) co-localization by immunofluorescence (DAPI: blue;). **p** Representative mIF images demonstrating expression of USP5 and CD8 in tumor cells. Scale bars: 10 μm(**o**) and 50 μm (**p**). Experiments were performed three times independently (**a**–**l, o**). Data were presented as mean ± SD (**a**, **i**,** l**, **o**). Groups were compared by two-tailed unpaired t-test (**a**,** j**). *P* < 0.05 was considered to be statistically significant

It is evident from the results above that FASN knockdown facilitated ferroptosis via blocking GPX4. We measured the mRNA level of GPX4 in the control and FASN-knockdown BC cells in order to investigate the molecular mechanism behind FASN-knockdown-driven ferroptosis. There is no discernible difference, as seen in Fig. 5a, indicating that any alterations in GPX4 take place at the protein level. Both FASN-knockdown and orlistat treatment accelerated the breakdown of GPX4 protein when protein synthesis was inhibited (Fig. 5b, c). To differentiate degradation routes, we also evaluated several protein degradation inhibitors, such as the corresponding lysosome and proteasome inhibitors chloroquine (CQ) and MG132. In orlistat-treated BC cells, CQ treatment was unable to reverse the decline in GPX4 (Fig. 5d, e).

To further explore the mechanism underlying this, we conducted LC–MS analysis and identified the deubiquitinase USP5 as a potential target of GPX4 (Fig. 5f, g). This deubiquitinase showed significantly higher expression in malignant versus adjacent normal tissues by mIF (Supplementary Fig. 6c, d). We first performed Co-IP experiments using the BC cells (Fig. 5h). Consequently, a strong binding between USP5 and GPX4 was found. In order to figure out the relationship between USP5 and GPX4, we conducted siRNA to knock down USP5 (Supplementary Fig. 6a, b). Herein, we validated that USP5 knockdown significantly restrained the expression of GPX4 at protein levels (Fig. 5i, j). Furthermore, with the knockdown of USP5, the binding between USP5 and GPX4 was reduced (Fig. 5k). As USP5 is a major regulator of the proteasome and possesses proteasome associated deubiquitination activity [45], ubiquitination assays indicated that the polyubiquitination of GPX4 was increased by USP5 knockdown in BC cells (Fig. 5l), demonstrating that USP5 stabilises the GPX4 protein by inhibiting its ubiquitin–proteasome degradation in BC cells. mIF and IF staining analysis indicated the colocalization of USP5 with GPX4 in the cytoplasm and nucleus of BC cells (Fig. 5m-o). Additionally, to investigate its relationship with CD8, we assessed their correlation and found that USP5 expression was negatively correlated with CD8 expression (Supplementary Fig. 6e). Moreover, cells that were positive for both USP5 and GPX4 also showed a negative correlation with CD8 expression (Supplementary Fig. 6f). This finding suggests that inhibiting ferroptosis is associated with a suppressed immune response, thereby hindering immunotherapy efficacy.

**Fig. 6:**
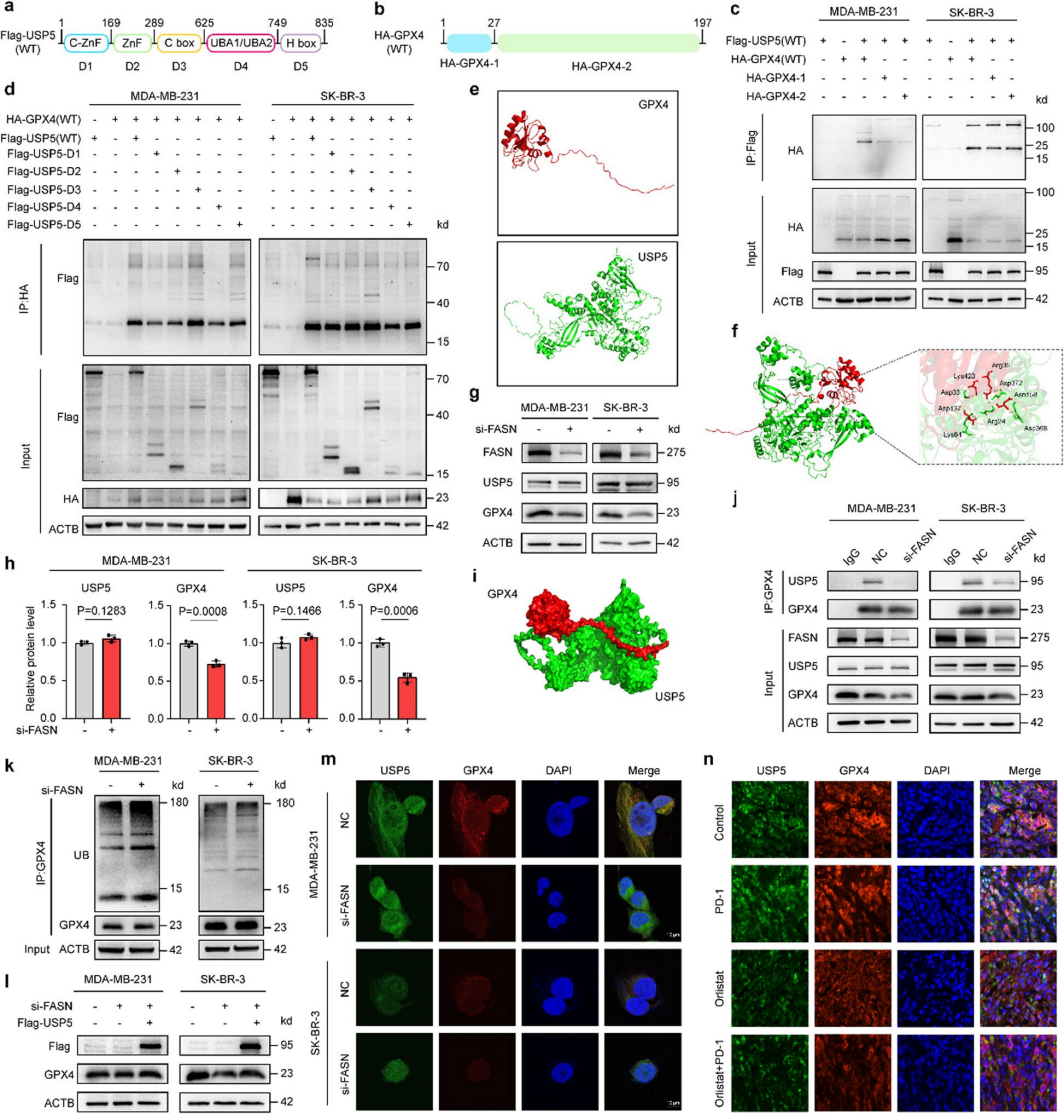
USP5 stabilizes GPX4 by deubiquitination in a FASN-dependent manner. **a** Schematic of USP5 and its truncation mutants. **b** Domain architecture of GPX4 and truncation mutants. **c** Co-IP in MDA-MB-231/SK-BR-3 cells co-transfected with HA-GPX4 (or mutants) and Flag-USP5 using anti-Flag antibody. **d** Reverse Co-IP with anti-HA antibody in cells expressing Flag-USP5 (or mutants) and HA-GPX4. **e** Cartoon model of GPX4-USP5 interaction. **f** Local interaction interface from molecular docking. **g**, **h** WB and quantification of USP5/GPX4 in FASN-KD cells. **i** Docking result (Surface + Cartoon mode). **j** IP-WB analysis in siFASN-expressing cells. **k** Ubiquitination assay in siFASN cells treated with MG132 (10 μM, 6 h). **l** GPX4 protein levels upon USP5 overexpression and FASN knockdown. **m** IF staining of endogenous GPX4/USP5 in FASN-KD cells. **n** Colocalization of ectopically expressed GPX4 and USP5. Scale bars: 10 μm(**m**) and 50 μm (**n**). Experiments were performed three times independently (**c**, **d**, **g**, **j**, **k**). Data were presented as mean ± SD (**g**). Groups were compared by two-tailed unpaired t-test (**g**). P < 0.05 was considered to be statistically significant

We next examined the interaction between USP5 and GPX4. USP5 consists of five individual domains designated C-ZnF, ZnF, C box, UBA1/UBA2 and H box [46]. To detect which domain(s) of USP5 were responsible for the interaction with GPX4, five truncated mutants were constructed (Fig. 6a). Co-IP results showed that USP5 mutants with only the C box domain has the ability to interact with GPX4 (Fig. 6d). These findings indicated the C box domain on USP5 is required for the interaction between USP5 and GPX4. Further, we constructed different truncated variants of GPX4. The full-length GPX4 protein contains 197 amino acids. We generated two fragments: one lacking amino acids aa28-197 (designated GPX4-Δ1, comprising 1–27) and another lacking amino acids 1–27 (designated GPX4-Δ2, comprising aa28-197). Both constructs were tagged with HA (Fig. 6b). Co-IP experiments with these two truncated GPX4 variants showed that both were able to interact with USP5 (Fig. 6c). These collective findings indicated that USP5 interacts with GPX4 through the C-box domains of USP5 and can bind to multiple regions across the GPX4 protein.

Subsequently, we used the method of protein–protein docking to predict the 3D complex model and potential interaction domains of USP5 and GPX4. The 3D spatial structures of these proteins were obtained from Uniprot databases (Fig. 6e), and the most likely complex model of USP5 and GPX4 binding was predicted using the HDOCK (Fig. 6f, i). The docking score, a more negative docking score indicates a more probable binding model, suggesting that a stable complex can be formed between them (confidence score: 0.774, Supplementary Table 1). PyMOL interaction interface analysis using a Python script was employed to map the “eyelash figure” of protein–protein interactions (Supplementary Fig. 8a, Supplementary Table 2), revealing the potential binding domains between USP5 and GPX4.

In the next study, we confirmed that endogenous USP5 coimmunoprecipitated with an endogenous GPX4 in FASN-knockdown BC cells (Fig. 6g, h, j), whereas FASN-knockdown group had the higher polyubiquitination of GPX4 than the control group (Fig. 6k). Moreover, overexpression of USP5 rescued the FASN knockdown-induced reduction of GPX4 (Fig. 6l). Besides, the colocalization of GPX4 and USP5 was weakened both in cells and tumors (Fig. 6m, n). Our findings indicate that FASN-knockdown promotes ferroptosis through the USP5/GPX4 axis in BC.

### FASN-Knockdown inhibits USP5 S-palmitoylation in BC

**Fig. 7:**
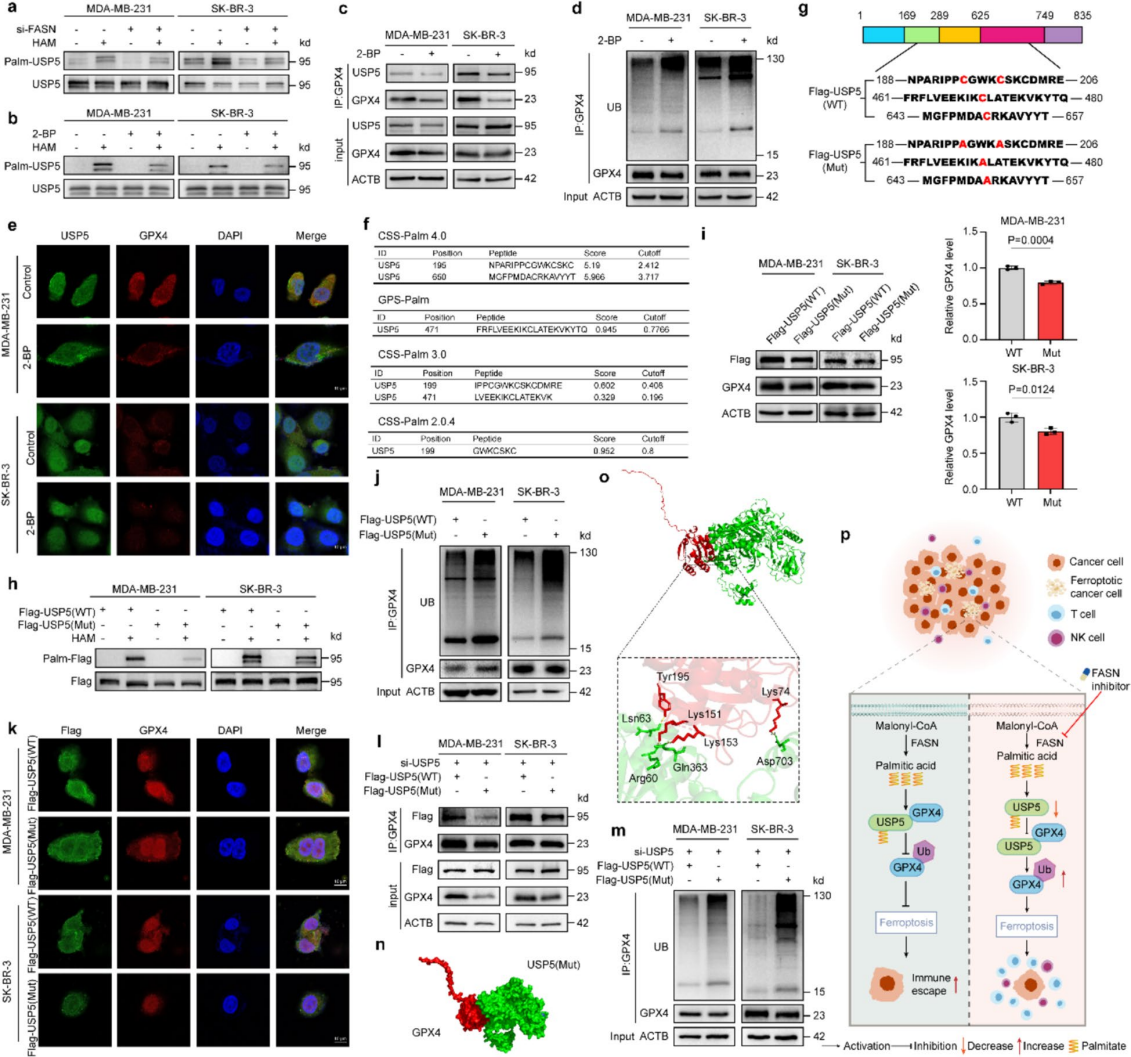
Palmitoylation of USP5 regulates GPX4 stability to suppress ferroptosis. **a** Breast cancer cells transfected with siRNA for 48 h were treated with MG132 (10 μM) for 6 h. USP5 palmitoylation was detected by acyl-biotin exchange (ABE) assay following immunoprecipitation (IP) with anti-USP5 antibody (hydroxylamine (HAM) removed palmitic acid (PA), free thiols labeled with BMCC-biotin, visualized by streptavidin-HRP). **b** Cells pretreated with 2-bromopalmitate (2-BP, 10 μM) for 48 h followed by MG132 (10 μM) for 6 h were subjected to ABE assay for USP5 palmitoylation (methods as in a). **c** GPX4 protein levels were analyzed by anti-GPX4 IP after 2-BP (10 μM) treatment for 48 h. **d** GPX4 ubiquitination was examined in 2-BP-pretreated (as in b) cells following MG132 treatment. **e** Immunofluorescence (IF) staining of USP5 (green) and GPX4 (red) in 2-BP-treated cells (nuclei: DAPI, blue). **f** Prediction of USP5 palmitoylation sites by GPS/CSS algorithms. **g** Schematic of wild-type (WT) USP5 and palmitoylation-deficient mutant (mut USP5). **h** ABE assay detecting palmitoylation of Flag-tagged WT/mut USP5 after MG132 treatment (IP with anti-Flag, methods as in a). **i** WB and quantification of Flag-GPX4 in WT/mut USP5-transfected cells. **j** GPX4 ubiquitination assay in WT/mut USP5-expressing cells treated with MG132. **k** IF staining of Flag (green) and GPX4 (red) in WT/mut USP5-transfected cells (nuclei: DAPI, blue). **l** Co-IP of GPX4 in USP5-knockdown cells rescued with WT/mut USP5. **m** GPX4 ubiquitination assay in rescue experiments (methods as in j). **n**,** o** Molecular docking models of GPX4-mut USP5 interaction (n: Surface + Cartoon representation; o: Key interfacial residues). **p** Working model: FASN promotes USP5 palmitoylation to enhance GPX4 binding, inhibiting GPX4 ubiquitination and subsequent degradation, thereby suppressing ferroptosis. Scale bars: 10 μm (**e**, **k**). Experiments were performed three times independently (**a-d**, **h**-**m**). Data were presented as mean ± SD (**i**). Groups were compared by two-tailed unpaired t-test (**i**). *P* < 0.05 was considered to be statistically significant

Interestingly, despite a reduction in the interaction between USP5 and GPX4 in cells with FASN knockdown, the levels of exogenously expressed USP5 remained unchanged (Fig. 6g). Given FASN's pivotal role in lipid metabolism [18, 47], we formulated the hypothesis that FASN might impede BC ferroptosis by modulating USP5 S-palmitoylation. Consistent with our hypothesis, a significant reduction in USP5 palmitoylation was observed in BC cells following FASN knockdown (Fig. 7a). Following treatment with exogenous palmitic acid, FASN failed to suppress the USP5 S-palmitoylation, which also failed to suppress the USP5-GPX4 interaction in FASN-knockdown cells (Supplementary Fig. 7a-c). Furthermore, ubiquitination assays revealed that exogenous palmitic acid decreased the polyubiquitination of GPX4 in BC cells (Supplementary Fig. 7d). The results confirmed that FASN inhibited USP5 S-palmitoylation by regulating palmitic acid. Additionally, the palmitoylation inhibitor 2-bromopalmitate (2-BP, 10 μM) effectively abolished USP5 S-palmitoylation to a level comparable to that of the control, yielding results similar to those observed with FASN knockdown (Fig. 7b-e).

Subsequently, we employed the palm acylation site software GPS-Palm and CSS-Palm for prediction (Fig. 7f). Then we synthesized wild-type overexpression plasmid (WT) and palmitoyl site mutation plasmid of USP5 (Fig. 7g). USP5 with point mutation did not undergo palmitoyl modification (Fig. 7h). Compared with the wild type, the expression of GPX4 was reduced in the mutant group (Fig. 7i). Mechanistically, ubiquitination assays demonstrated enhanced polyubiquitination of GPX4 in BC cells expressing the USP5 mutant (Fig. 7j). Additionally, IF showed weakened colocalization between GPX4 and USP5 (Fig. 7k). Next, we transfected cells with USP5 knockdown with plasmids encoding wild-type and mutant USP5. The results showed that, compared with the USP5-WT group, the binding between USP5 and GPX4 was weakened in the mutant group (Fig. 7l). Additionally, the ubiquitination of GPX4 was increased (Fig. 7m).

Additionally, we predicted the potential interactions of USP5-WT and the USP5 mutant (Supplementary Fig. 8b) with GPX4, respectively. According to the docking score, the USP5-WT group exhibited stronger binding ability with GPX4 compared to the USP5 mutant group (Fig. 7n, o, Supplementary Fig. 8c, d, Supplementary Table 3). Collectively, these results suggest that FASN knockdown promotes ferroptosis by inhibiting USP5 S-palmitoylation in BC cells (Fig. 7p).

## Discussion

Our study advances the understanding of the mechanisms by which FASN affects GPX4 regulation of ferroptosis. We identified USP5 as a deubiquitinase for GPX4, which stabilizes GPX4 through direct interaction and deubiquitination. Beyond ubiquitination, FASN-mediated S-palmitoylation of USP5 is critical, as it enhances the binding between USP5 and GPX4, thereby reinforcing GPX4-dependent suppression of ferroptosis. Prior to this work, it remained unclear whether USP5 could undergo palmitoylation or whether USP5 could bind to and stabilize GPX4. In addition, we found that the FASN inhibitor orlistat enhances tumor sensitivity to immunotherapy by promoting ferroptosis.

Immunotherapy has emerged as a crucial treatment modality for patients with BC, particularly those with TNBC [48]. However, a significant proportion of patients experience residual tumors and local recurrence due to immune resistance, while others exhibit low sensitivity to immunotherapy [49–51]. The underlying mechanisms contributing to these therapeutic failures are multifaceted, including immunosuppression within the TME, variations in immune cell subtype distribution, DNA repair mechanisms, and TMB [52–54]. But the problems associated with therapy failure have not been adequately addressed by our current understanding of these systems. Therefore, investigating alternative approaches to enhance therapy response is essential. Our study demonstrates that orlistat, a known FASN inhibitor, exhibits synergistic antitumor effects when combined with anti-PD-1 therapy. At low doses, orlistat significantly delayed tumor growth and enhanced CD8^+^ T cell infiltration in the tumor microenvironment, thereby improving the efficacy of anti-PD-1 immunotherapy across mouse tumor models. However, the mechanisms underlying the sensitization of immunotherapy by FASN inhibitors require further investigation.

In recent studies, ferroptosis has a certain degree of crosstalk with the immune system in hepatocellular carcinoma, BC, and lung cancer [55–57]. FASN, a key enzyme that regulates the rate of fatty acid synthesis, significantly contributes to tumor proliferation and resistance to treatments. In our study, we demonstrated that FASN knockdown inhibits BC growth and metastasis, consistent with previous reports on FASN's role in tumor progression [58]. Furthermore, we uncovered a significant relationship between FASN and ferroptosis in BC cells, with FASN knockdown-induced cell death being rescued by the ferroptosis inhibitor ferrostatin-1. However, the precise mechanisms underlying FASN-mediated regulation of ferroptosis remain to be elucidated. Based on the results of our PCR and WB results, it was implied that the downstream target of FASN was GPX4.

As a glutathione peroxidase, GPX4 converts lipid hydroperoxides into lipid alcohols, ultimately eliminating lipid peroxidation [59]. A number of studies have shown that GPX4 is an important protein during ferroptosis [60]. Yang, F. et al. have reported that inhibition of GPX4 not only induces ferroptosis in tumors but also enhances anti-tumor immunity. The combination of GPX4 inhibitors with anti-PD1 therapy shows superior efficacy compared to monotherapy [11]. PUFAs are the lipids that are most prone to peroxidation during ferroptosis, while FASN inhibition is considered to lead to the accumulation of PUFA in phospholipids, particularly in phosphatidylcholine (PC) [61]. Based on these findings, we focused on the regulation of GPX4 to clarify the mechanisms underlying FASN-knockdown-induced ferroptosis.

USP5, also named as isopeptidase T (IsoT), belongs to the peptidase C19 family and prefers to cleave unanchored polyubiquitin chains [62], and plays a crucial role in various cellular processes, such as DNA repair and protein stabilization and localization. Pan, J. et al. reported that USP5 directly interacted with PD-L1 and deubiquitinated PD-L1, therefore enhancing PD-L1 protein stability [63]. In our experiments, we confirmed the binding of USP5 and GPX4. However, compared with the control group, the total protein expression of USP5 did not change in the FASN knockdown group. This inevitably reminds us of the close relationship between FASN and S-palmitoylation. Interestingly, S-palmitoylation promotes the stability of USP5. Through comprehensive analysis of the results predicted by the GPS-Palm databases, we determined that the S-palmitoylation of USP5 affects its binding to GPX4. This finding underscores the importance of USP5 in FASN-regulated ferroptosis.

Furthermore, in our study, we observed that treatment with orlistat in vitro did not promote apoptosis in BC cells to the same extent as FASN knockdown. This discrepancy may be attributed to the possibility that FASN regulates apoptosis through functions independent of its lipogenic activity, such as activating the TLR3/IRF7 pathway [64]. Additionally, differences in the potency and duration of FASN inhibition between the two approaches may play a role. This observation also suggests that, in combination therapies involving FASN inhibitors, careful consideration of dosing and treatment duration is required. In addition, our study did not identify the specific mechanism by which FASN regulates the S-palmitoylation of USP5; this will be further investigated in subsequent research.

Overall, our study elucidated the specific and detailed mechanism of how FASN is involved in the modulation of ferroptosis in BC cells. The present study demonstrated that inhibition of FASN expression promotes ferroptosis by decreasing the binding of USP5 to GPX4, which in turn exhibits a synergistic effect on inhibiting the proliferation of BC cells. More notably, we also found that orlistat, an inhibitor of FASN, exhibited combination therapeutic efficacy with anti-PD-1 in vivo, thereby laying a solid foundation for future clinical applications.

## Methods

### Bioinformatics analysis

We downloaded transcriptomic sequencing (RNA-seq) data and corresponding clinical information from the BRCA dataset (1,069 BC patients with complete survival records) of The Cancer Genome Atlas (TCGA) from UCSC Xena (https://xena.ucsc.edu/public). Besides, Single-cell RNA sequencing (scRNA-seq) data of BC (GSE180286) were downloaded from the Gene Expression Omnibus (GEO) database (https://www.ncbi.nlm.nih.gov/geo/), with sequencing performed on the HiSeq X Ten platform. Only primary tumor samples were retained, comprising a total of 5 cases. Moreover, gene expression profiles with accession numbers GSE194040 and GSE173839 were downloaded from the GEO database, retaining only breast cancer patients who received immunotherapy and had documented treatment outcomes. The cohorts consisted of 95 cases from GSE194040 and 72 cases from GSE173839. Subsequently, the "removeBatchEffect" function from the "limma" R package was applied to merge the datasets while eliminating batch effects. Gene set enrichment analyses (including ssGSEA [65] and conventional GSEA [66]) were performed to evaluate functional associations of gene clusters. Differential expression analysis and functional enrichment were conducted with the "FindAllMarkers" algorithm and "enrichR" tool, respectively.

### Single-cell transcriptomic data preprocessing and integration

The scRNA-seq data in dataset GSE180286 were downloaded and analyzed using the Seurat R package [67] (v4.0.4, http://satijalab.org/seurat/). The preprocessing pipeline included data quality control, gene/cell filtering, normalization, highly variable gene identification, and data scaling. To account for variations across samples and experimental techniques, we used the “RunHarmony” function from the R package harmony to minimize batch effects [68]. Based on the top 4,000 variable genes, we performed principal component analysis (PCA) and reduced the dimensionality of the scaled integrated data matrix to a two-dimensional space using the first 20 principal components, followed by visualization with t-distributed stochastic neighbor embedding (t-SNE).

### Cell type identification and immune microenvironment characterization

We annotated cell types using validated cell type-specific expressed genes from the literature, including tumor cells, B cells, T cells, macrophages, endothelial cells, and fibroblasts. Based on the TCGA-BRCA data, we combined feature gene sets and algorithms from previous studies to estimate the immunological features of the tumor microenvironment (TME) for each patient, including 122 immune regulatory genes, effector genes of tumor-infiltrating immune cells (TIICs), and 18 T cell inflammation-related specific genes and their weighted coefficients [69, 70]. Then, we used the ESTIMATE algorithm to assess tumor purity, immune cell infiltration abundance, and stromal cell proportion [71], and comprehensively calculated the relative abundance of TIICs using independent algorithms including xCell [72], EPIC [73], MCP_counter [74], and TISIDB [75]. We also collected a feature set of 29 immune cell types and immune-related pathways from previous studies and used the ssGSEA algorithm to infer the scores of various immune cells and related pathways.

### Multiplex and single-plex immunofluorescence

A paraffin-embedded tissue microarray (TMA) (catalog HBre-Duc159Sur-01) was obtained from Outdo BioTech (Shanghai, China). The primary antibodies used for immunofluorescence staining were: FASN (catalog 3180, CST, USA), USP5 (10,473–1-AP, Proteintech, China), GPX4 (67,763–1-Ig, Proteintech, China) and CD8 (catalog 85,336, CST, USA).

Cell samples were fixed with 4% paraformaldehyde (PFA) in PBS for 15 min at room temperature (RT), followed by permeabilization with 0.1% Triton X-100 (Solarbio, T8200) in PBS for 20 min at RT. Non-specific binding sites were blocked with 10% normal goat serum (NGS) in PBS for 1 h at RT. Primary antibodies were diluted in blocking buffer and incubated with samples overnight at 4 °C, including anti-USP5 (10,473–1-AP, Proteintech, China) and anti-GPX4 (67,763–1-Ig, Proteintech, China). After PBS washes, samples were incubated with species-matched Alexa Fluor-conjugated secondary antibodies for 1 h at RT protected from light. Nuclei were counterstained with DAPI (C1002, Beyotime, China) for 5 min. Confocal images were acquired using a Nikon A1 + laser scanning microscope (NIS-Elements AR software) with a 60 × oil-immersion objective, maintaining identical acquisition parameters across compared samples.

### Immunohistochemistry assay

The tissue microarray of human BC (catalog HBreD090Bc03) was acquired from Outdo Biotech Company (Shanghai, China). Immunohistochemical (IHC) staining was performed on formalin-fixed, paraffin-embedded (FFPE) breast cancer tissue sections following standard protocols. Briefly, sections were deparaffinized in xylene and rehydrated through a graded ethanol series (100% to 70%). Antigen retrieval was conducted in 0.01 M citrate buffer (pH 6.0) at 98 °C for 20 min. After blocking with 5% BSA for 20 min at room temperature, sections were incubated with primary antibodies (FASN, 10,624–2-AP, Proteintech, China; CD8, 66,868–1-Ig, Proteintech, China) overnight at 4 °C. Following PBS washes, sections were incubated with HRP-conjugated secondary antibody for 20 min at room temperature. DAB substrate was applied for chromogenic development, followed by hematoxylin counterstaining. Sections were dehydrated through graded ethanol, cleared in xylene, and mounted with resin. Immunoreactivity was evaluated using an immunohistochemical reactivity score (IRS) calculated as the product of staining intensity (0–3) and percentage of positive cells (0–4).

### Cell culture and transfection

Human BC cells SK-BR-3 (CL-0211, Procell, China) and MDA-MB-231 (catalog KGG3220-1, KeyGEN Biotech, China) were cultured in Dulbecco’s minimum essential medium (KGM12800N-500, KeyGEN Biotech, China) and Leibovitz's L-15 medium (KGL1802-500, KeyGEN Biotech, China), respectively, supplemented with 10% fetal bovine serum (10,091,148, Thermo Fisher Scientific, USA) and 1% penicillin streptomycin solution (KGL2303-100, KeyGEN Biotech, China). All cells were grown in a cell incubator under 5% CO2 and 37 °C.

For transfection experiments, small interfering RNA (siRNA) oligonucleotides (KeyGEN Biotech, China) or plasmid DNAs (Zebrafish Biotech, China) were transfected into the cells using Lipofectamine 3000 transfection reagent (L3000015, Thermo Fisher, USA) according to the manufacturer’s instructions. The specific sequences of the siRNA oligonucleotides are shown in Supplementary Table 4.

### RNA isolation and qRT–PCR

The total RNA extraction was performed using RNAeasy Isolation Reagent (No. RC112-01, Vazyme, China). qRT–PCR was performed using a HiScript III RT SuperMix for qPCR (+ gDNA wiper) (No. R323-01, Vazyme, China) and ChamQ Universal SYBR qPCR Master Mix (No. Q711-02, Vazyme, China) according to the manufacturer’s instructions. All procedures were performed according to the manufacturer’s instructions. Total RNA concentrations were measured with a NanoDrop2000, and an equal amount of RNA from each sample was used for cDNA synthesis using a RevertAid cDNA synthesis kit. The cDNAs were subsequently used for qPCR analysis with Power SYBR Green Master Mix using the Step One Plus Real-Time PCR System. The specific primer sequences for qPCR were listed in Supplementary Table 5.

### Immunoblotting and Co-immunoprecipitation (Co-IP)

Cell lysates were separated by SDS-PAGE, followed by wet transfer onto 0.45 μm PVDF membranes, which were then blocked with 5% BSA in PBST solution for 30 min. As for IP experiments, the specific antibodies were mixed with Protein A/G Magnetic Beads (HY-K0202, MCE, USA) for 2 h at 4 °C. Then the beads were collected and incubated with antigen sample for 2 h at 4 °C. After which four times washes with PBST were operated. Finally, the loading buffer was added into beads and the mix ture was boiled at 100 °C for 10 min for further experiments. The antibodies used were as follows: anti-HA (51,064–2-AP, Proteintech, China), anti-FLAG (20,543–1-AP, Proteintech, China), anti-USP5 (Proteintech, China), anti-FASN (ab128856, Abcam, UK), anti-GPX4 (ab125066, Abcam, UK).

### Proliferation assays

Pretreated cells were resuspended and seeded into 96-well platesat a density of 2 × 10^^3^ cells per well for CCK-8 assays. Cell proliferation was quantified by measuring the absorbance at 450 nm. Additionally, cells were evenly seeded into six-well plates with a seeding density of 5 × 10^^3^ cells per well and cultured for 10 days in a standard cell culture incubator. Colonies were subsequently fixed with paraformaldehyde (P1110, Solarbio, China) and stained using crystal violet dye (G1061, Solarbio, China). Finally, photography was employed to capture the samples.

### Transwell migration and invasion assays

Cells were seeded in a 6-well plate and then transfected with siRNA or orlistat (50 μM, MCE, China). For Transwell migration assays, a total of 2 × 10^^4^ cells in 200 μl of serum-free medium were seeded in the upper chamber of 8-μm Transwell inserts (3422, BD Biosciences, USA). The lower chamber was filled with 10% FBS medium. Twenty-four hours later, the remaining cells in the upper chamber were removed. The cells adhering to the membrane were fixed with methanol for 15 min and then stained with 0.1% crystal violet (G1061, Solarbio, China) for 6 min. For the invasion assays, the upper chamber was precoated with 80 μl of Matrigel (356,234, BD Biosciences, USA), and 2 × 10^^5^ cells in 200 μl of serum-free medium were seeded. The rest of the procedure was the same as that for the migration assays.

### GSH, MDA, and SOD content measurement

Levels of glutathione (GSH), superoxide dismutase (SOD), and malondialdehyde (MDA) were determined utilizing specific assay kits. Specifically, the GSH kit (A006–1–1) and MDA kit (A003–1–2) were sourced from the Nanjing Jiancheng Bioengineering Institute, while the SOD kit (E-BCK020-M) was obtained from Elabscience.

### Lipid peroxidation measurement

To measure cellular lipid peroxidation, the BC cells were treated as indicated exposure, then loaded with 5 μM of the oxidation-sensitive probe C11-BODIPY581/591 (D3861, Thermo, USA) for 20 min, followed by staining with DAPI.

### Iron content measurement

Iron Colorimetric Assay Kit were purchased from (E-BC-K139-S) Elabscience. First, we collected the BC cells and treated supernatant with iron coloring solution, boiling water bath for 5 min, cooling in running water, and centrifuged the mixture at 2300 g for 10 min. Then, we transferred the supernatant to a new tube for further absorbance analysis at 520 nm.

### Protein degradation analysis

Pretreated cells were preincubated with cycloheximide at a concentration of 10 μM. After a 1-h incubation period, the BC cells were subjected to the designated treatment conditions. The expression of GPX4 in both control and treatment cells was evaluated using a Western blotting assay.

### Immunoprecipitation coupled with mass spectrometry assay

The antigen–antibody-magnetic bead complex was obtained as described above. Cell lysates were separated by SDS-PAGE. The samples were subjected to in-gel digestion followed by destaining, reduction, alkylation, and tryptic digestion. The resulting peptides were desalted using C18 cartridges prior to LC–MS/MS analysis. Peptide separation was performed on an Easy nLC system with mobile phase A (0.1% formic acid in water) and B (0.1% formic acid in 80% acetonitrile), using a 60-min linear gradient from 0 to 100% B. Mass spectrometric analysis was conducted on a Q Exactive Plus instrument in positive ion mode, with full MS scans (350–1800 m/z) at 70,000 resolutions, followed by HCD fragmentation of the top 10 most intense precursors. Raw data were processed using Proteome Discoverer 2.2 and searched against databases using MASCOT 2.6, with identification confidence filtered at FDR < 1%.

### Immunoprecipitation‑acyl‑biotin exchange

The IP-ABE was performed following the recommendations of the IP-ABE Palmitoylation Kit for western blot (Aimsmass, China). Briefly, cells were lysed and subsequently centrifuged prior to overnight immunoprecipitation with agarose beads and specific antibodies. After three washes, the precipitates were divided equally into two portions: one portion served as the HAM-control, while the other half underwent HAM + treatment at room temperature for 60 min. The precipitate was gently washed and subsequently incubated with BMCC–biotin buffer for 60 min. Following washing steps, samples were analyzed through western blot analysis, where palmitoylated USP5 was detected using HRP-conjugated streptavidin provided in the kit. The antibodies employed in this investigation included anti-USP5 (10,473–1-AP, Proteintech, China) and anti-FLAG (20,543–1-AP, Proteintech, China).

### Protein–protein docking

Protein–protein docking analysis between USP5 (AlphaFoldDB: O75300) and GPX4 (AlphaFoldDB: P36969) was performed using HDOCK [76] software. Prior to docking, energy minimization of the protein structures was conducted using the protein preparation wizard tool in PyMOL 2.5.5 [77]. The docking visualization was rendered with PyMOL 2.5.5, while 2D interaction diagrams were generated using LigPlot + 2.2.8 [78].

### Animal experiment

Female BALB/c mice (4–6 weeks old) from the Shanghai Laboratory Animal Center (Shanghai, China) were housed in specific pathogen-free facilities at 20–24℃ under 12-h light/dark cycles. The murine breast cancer cell line 4T1 (KG338, KeyGEN Biotech, China) was cultured in Dulbecco’s minimum essential medium (KGM12800N-500, KeyGEN Biotech, China) supplemented with 10% fetal bovine serum (10099141C, Gibco, USA) at 37℃ with 5% CO2. All cell lines were free from mycoplasma and authenticated recently by short tandem repeat profiling. The mouse breast cancer model was established by subcutaneously injecting approximately 5 × 10^^6^ cells into each BALB/c mouse. Tumor size was monitored every 2–3 days using calipers, and tumor volume (V) was calculated using the formula V = (length × width^2^) / 2.

Upon tumors reaching an average size of approximately 100 mm^3^, tumor-bearing mice were randomly assigned to groups. The control group received oral administration of phosphate-belanced solution (PBS). The orlistat group received daily intraperitoneal injection of orlistat (HY-B0218, MedChemExpress, Shanghai, China) at 240 mg/kg. The anti-PD-1 group was injected intraperitoneally with the neutralizing antibody InVivoMAb antimouse PD-1 (BE0273, BioXCell, Lebanon, NH, USA) at 200 µg/mouse three times a week. The combination group received a daily intraperitoneal injection of the orlistat at 240 mg/kg. And intraperitoneal injections of the anti-PD-1 antibody at 200 µg/mouse three times a week. The tumors were removed from the unconscious animals at day 21 after the initiation of the treatment, which was subsequently documented and weighed.

### Statistical analysis

All analyses were performed using R 4.4 or GraphPad Prism 9.5. Continuous variables were analyzed by Student's t-test (normal distribution) or Wilcoxon rank-sum test (non-normal distribution), while categorical variables were assessed with Fisher's exact test. One-way ANOVA was applied for multi-group comparisons of normally distributed data. Statistical significance was set at *P* < 0.05.

## Supplementary Information

Supplementary Material 1

Supplementary Material 2

## Data Availability

No datasets were generated or analysed during the current study.
