# Supplementary material for: FASN inhibits ferroptosis in breast cancer via USP5 palmitoylation-dependent regulation of GPX4 deubiquitination
Source: J Exp Clin Cancer Res. 2025 Oct 14;44:289. doi: 10.1186/s13046-025-03548-8 (PMC12523187; doi:10.1186/s13046-025-03548-8)
Supplement: Supplementary file 1 — Supplementary Material 1 [file 13046_2025_3548_MOESM1_ESM.docx]

**
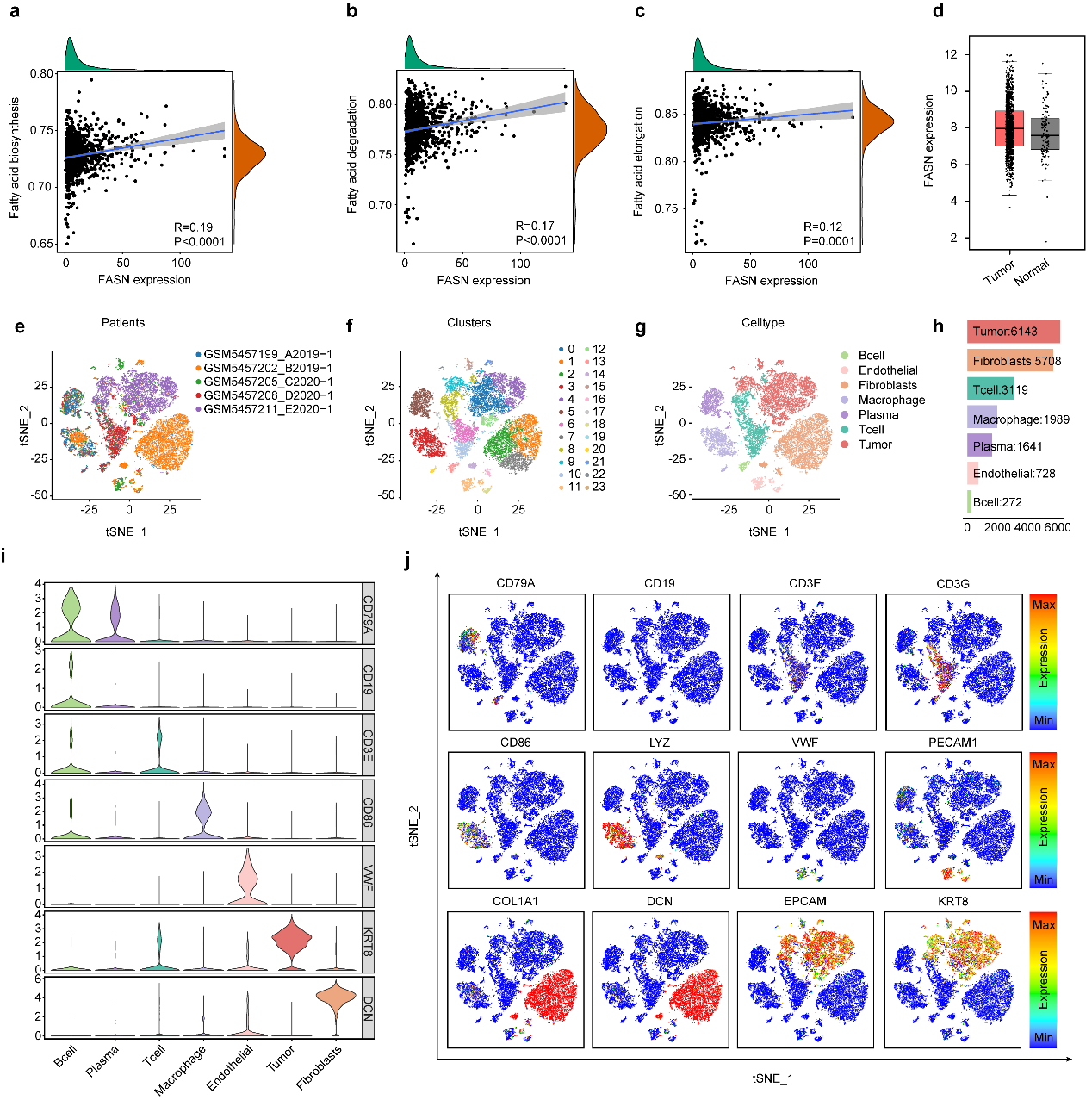
**

**Supplementary fig. 1 FASN links fatty acid metabolism to breast cancer progression through single-cell transcriptomic profiling.**

**a-c** Association between FASN and fatty acid metabolism pathways including fatty acids biosynthesis (**a**), fatty acid degradation (**b**) and fatty acid elongation (**c**). **d** Differential expression of FASN between normal breast tissues and breast invasive carcinoma analyzed by GEPIA2. **e** t-SNE visualization of integrated scRNA-seq datasets after batch effect correction, with samples indicated by distinct colors. **f** High-resolution unsupervised clustering identifies 24 initial cell clusters. **g-i** Annotation of 7 major cell types: t-SNE plot color-coded by cell type identity (**g**), bar plot quantifying cell numbers per type (**h**), violin plots showing expression levels of canonical marker genes across types (**i**). **j** t-SNE validation of cell type-specific gene expression patterns. Groups were compared by Wilcoxon rank-sum test (d). P < 0.05 was considered to be statistically significant. t-SNE, t-distributed stochastic neighbor embedding; scRNA-seq, single-cell RNA sequencing.

**
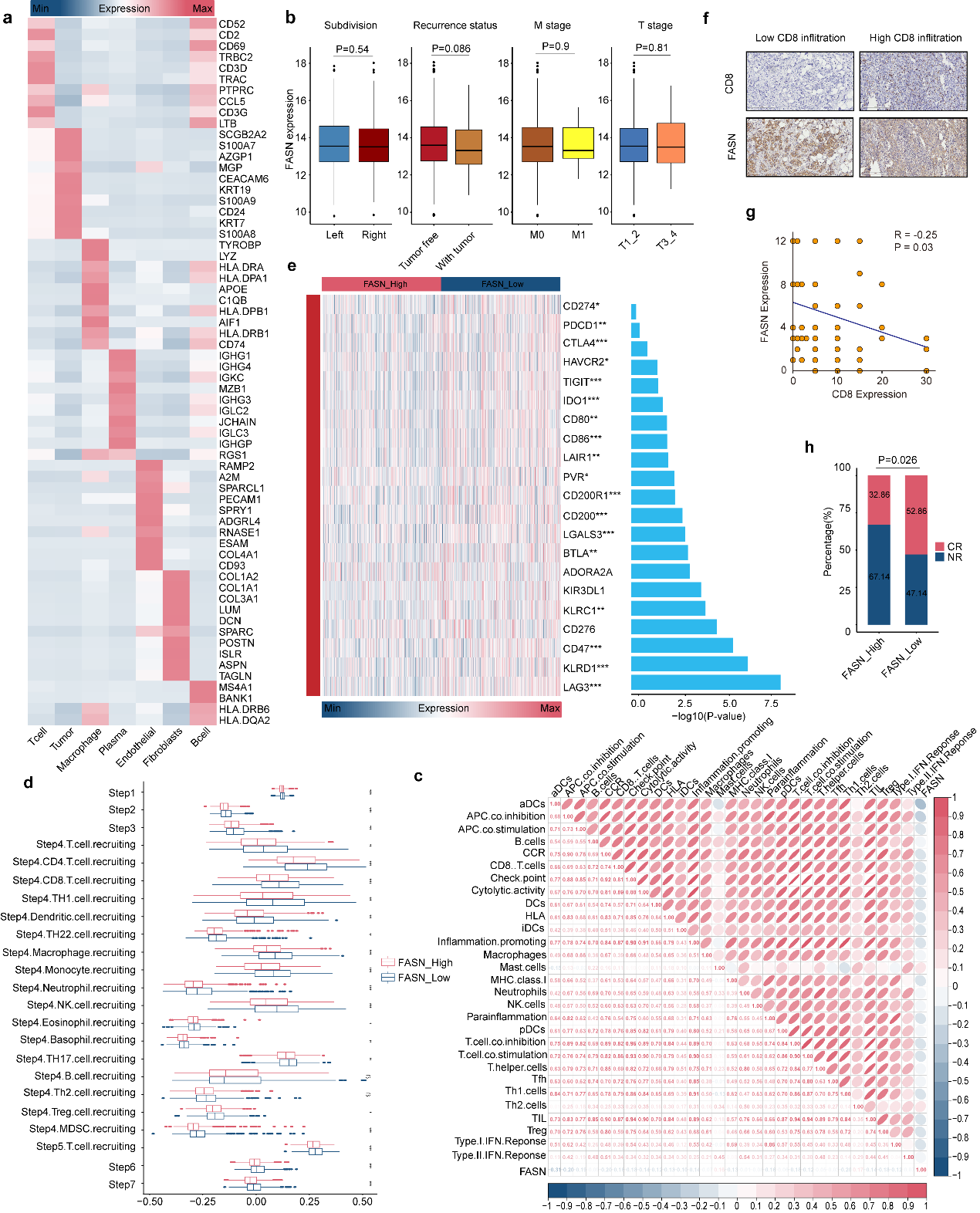
**

**Supplementary fig. 2 FASN orchestrates immunosuppressive microenvironment and clinical outcomes in breast cancer.**

**a** Heatmap displaying top 10 signature genes per cell type. **b** TCGA-BRCA analysis of FASN expression across clinicopathological features (boxplot elements: median line.). **c** Correlation heatmap depicting relationships between FASN expression and 29 immune cell types/immune activation pathways. **d** Boxplots comparing cancer immunity cycle activity scores between FASN-high and FASN-low groups (stratified by median line). **e** Heatmap (left) and significance bars (right) of immune checkpoints in FASN-high/low groups (TCGA-BRCA, median split). **f**, **g** Representative immunohistochemistry (IHC) images demonstrating expression of FASN and CD8 in tumor cells (**f**) with Pearson correlation analysis (**g**). **h** Stacked bars show CR/NR proportions in FASN-high/low groups (median split, Fisher's exact test). Groups were compared by Wilcoxon rank-sum test (**b**, **d**) or Fisher's exact test (**h**). P < 0.05 was considered to be statistically significant. CR: complete remission; NR: no remission.


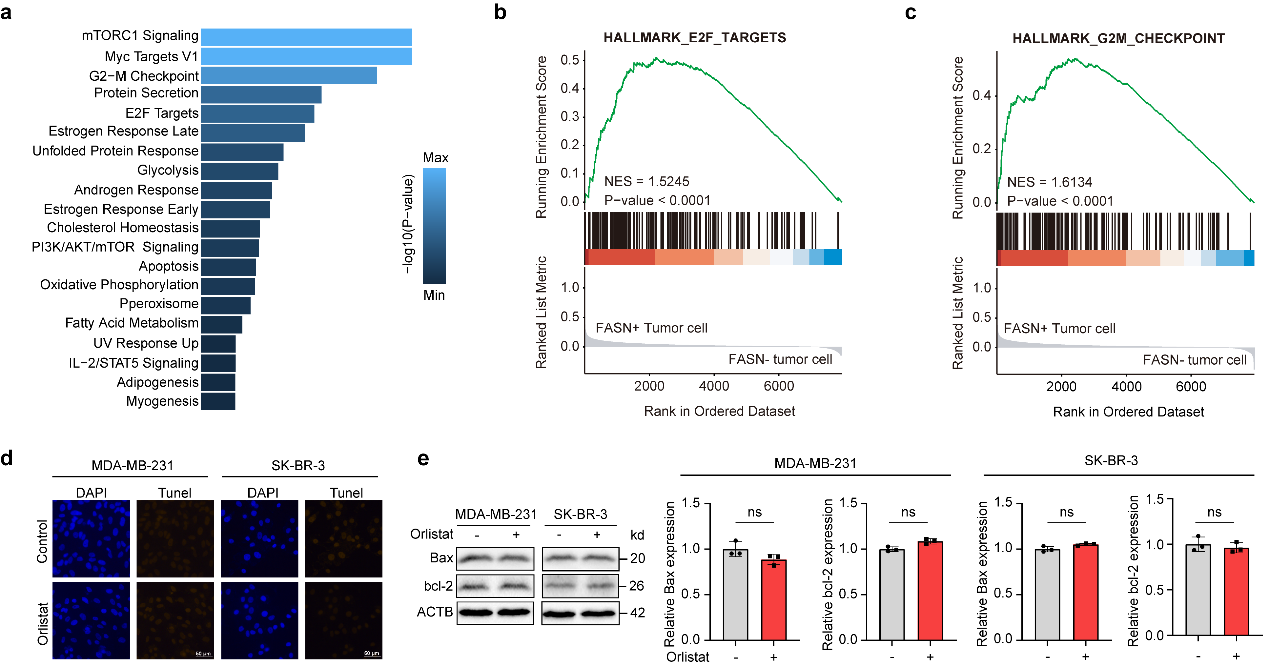


**Supplementary fig. 3 Functional characterization of FASN-high tumor cells and pharmacological inhibition effects.**

**a-c** Single-cell transcriptomic analysis of breast cancer: Bar plot showing significantly enriched HALLMARK pathways in FASN+ tumor cells (**a**). Dot plots demonstrating correlations between FASN expression and proliferation-related HALLMARK pathways (**b**, **c**). **d** TUNEL assay showing apoptosis induction after 48-hour orlistat treatment (blue: DAPI-stained nuclei; red: TUNEL-positive cells). **e** Western blot analysis of apoptosis markers (Bax/bcl-2) in orlistat-treated cells with β-actin loading control (left). Quantified band intensities are shown (right). Scale bars: 50 μm (**d**). Experiments were performed three times independently (**d**, **e**). Data were presented as mean ± SD (**e**). Groups were compared by two-tailed unpaired t-test (**e**). P < 0.05 was considered to be statistically significant.


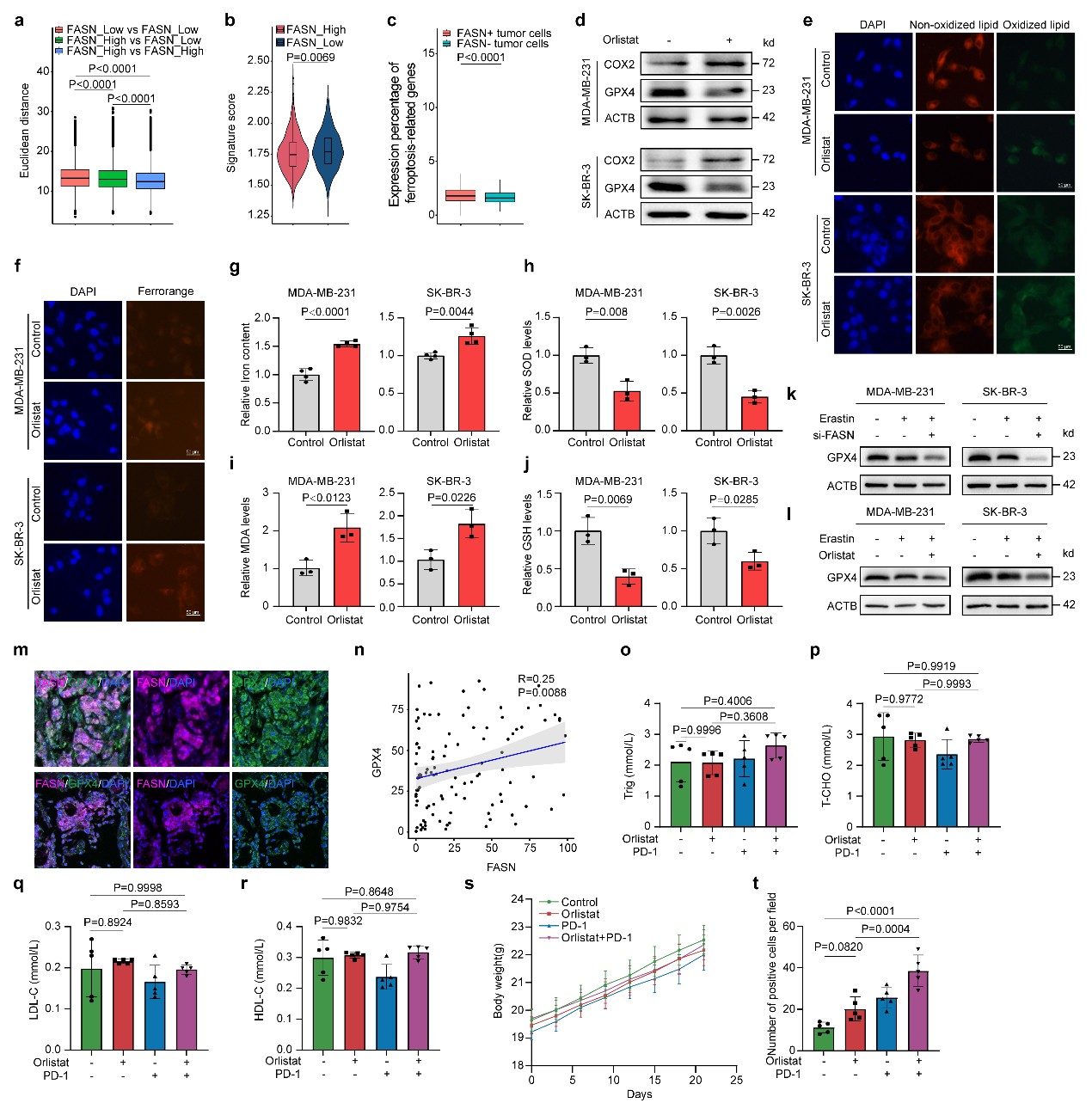


**Supplementary fig. 4 FASN-associated ferroptosis features and therapeutic effects of orlistat in breast cancer models.**

**a-b** TCGA-BRCA cohort analysis: uclidean distance analysis of ferroptosis-related gene expression between FASN-high (blue) and FASN-low (red) groups (green: inter-group distances) (**a**). ssGSEA scores of ferroptosis pathways comparing FASN-high vs. FASN-low groups (**b**). **c** scRNA-seq analysis: ferroptosis activity scores in FASN+ vs. FASN- tumor cells. **d** Western blot analysis of ferroptosis markers (GPX4/COX2) in orlistat-treated cells with β-actin loading control. **e** Lipid peroxidation detection in orlistat-treated MDA-MB-231 and SK-BR-3 cells using C11-BODIPY 581/591 probe. Representative fluorescence images show oxidized lipid (green) and non-oxidized lipid (red). **f, g** Orlistat elevates intracellular ferrous iron (Fe²⁺) levels in MDA-MB-231 and SK-BR-3 cells, as quantified by FerroOrange fluorescence (**f**, 5 μM probe) and ferrozine assay(**g**). **h-j** Impact of orlistat on oxidative stress and ferroptosis in MDA-MB-231 and SK-BR-3 cells. Cells were treated as in (**d**), then relative SOD (**h**), MDA (**i**) and GSH (**j**), content were detected by their corresponding kits. **k**, **l** FASN inhibition synergizes with erastin to induce ferroptosis. **m**, **n** Representative multiplex immunofluorescence (mIF) images demonstrating expression of FASN and GPX4 in tumor cells (**m**) with Pearson correlation analysis (**n**). **o-r** Blood lipid profiling: Serum triglycerides, total cholesterol, LDL-C, and HDL-C levels across treatment groups. **s** Body weight changes in 4T1-tumor bearing mice receiving orlistat/anti-PD-L1 combo therapy. **t** The number of CD8-positive cells per field. Scale bars: 50 μm (**e**, **f**). Experiments were performed three times independently (**d**-**j**). Data were presented as mean ± SD (**g-j**, **m**, **n**). Groups were compared by Wilcoxon rank-sum test (**a**-**c**), two-tailed unpaired t-test (**g**-**j**) or one-way ANOVA followed by Fisher’s LSD test (**m**-**t**). P < 0.05 was considered to be statistically significant.


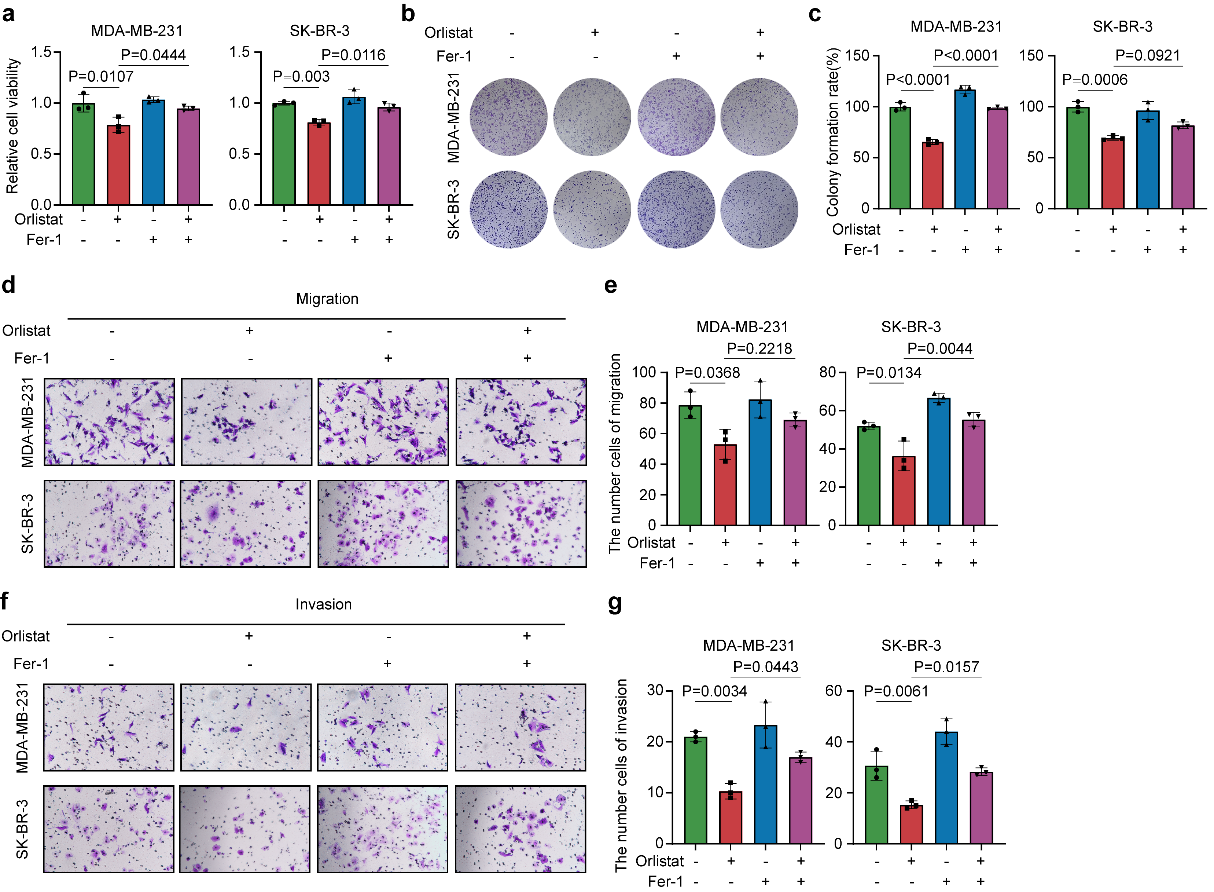


**Supplementary fig. 5** **Therapeutic effects of orlistat and ferrostatin-1 (Fer-1) combination treatment on breast cancer cell malignant phenotypes.**

**a-g** Effects of Orlistat combined with ferrostatin-1 (Fer-1, 5 μM) on malignant behaviors of breast cancer cells, including cell viability (**a**), Clonogenic survival (**b**, **c**), migration (**d**, **e**) and invasion (**f**, **g**). Scale bars: 50 μm (**d**, **f**). Experiments were performed three times independently (**a**–**g**). Data were presented as mean ± SD (**a**, **c, e**, **g**). Groups were compared by one-way ANOVA followed by Fisher’s LSD test (**a**, **c, e**, **g**). P < 0.05 was considered to be statistically significant.

**
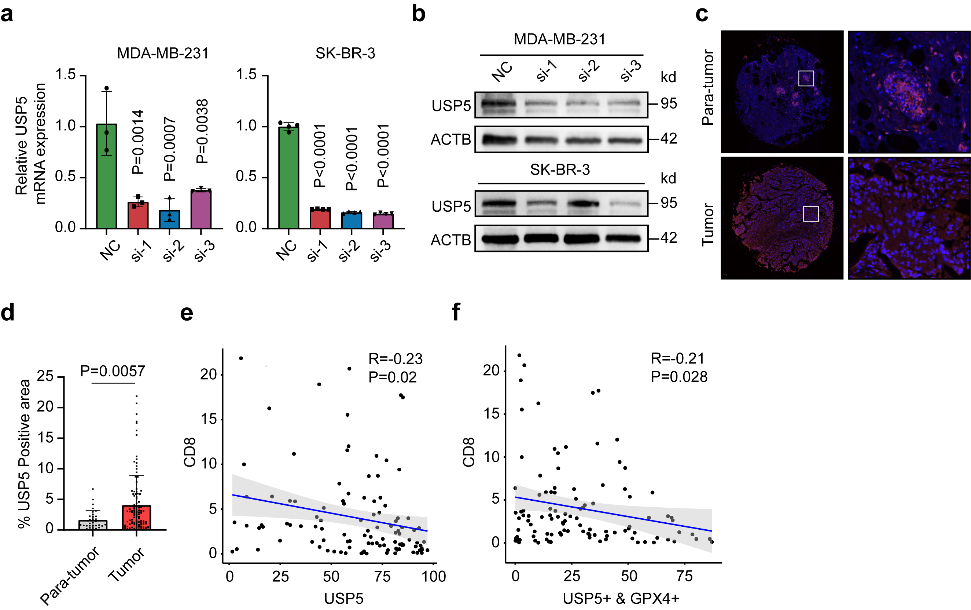
**

**Supplementary fig. 6** **USP5 regulates GPX4 co-expression and CD8+ T cell infiltration.**

**a**, **b** USP5 mRNA (**a**, qRT-PCR) and protein (**b**, WB) levels in siRNA-transfected cells. **c** Multiplex immunofluorescence (mIF) of USP5 (red) with DAPI (blue). **d** Quantification of USP5+ positive area fraction. **e** Quantitative multiplex immunofluorescence analysis of USP5 correlation with CD8. **f** Quantitative multiplex immunofluorescence analysis of GPX4 and USP5 co-expression correlation with CD8. Scale bars: 50 μm (**c**). Experiments were performed three times independently (**a**, **b**). Data were presented as mean ± SD (**a**, **b**, **d**). Groups were compared by two-tailed unpaired t-test (**d**) or one-way ANOVA followed by Fisher’s LSD test (**a**, **b**). P < 0.05 was considered to be statistically significant.


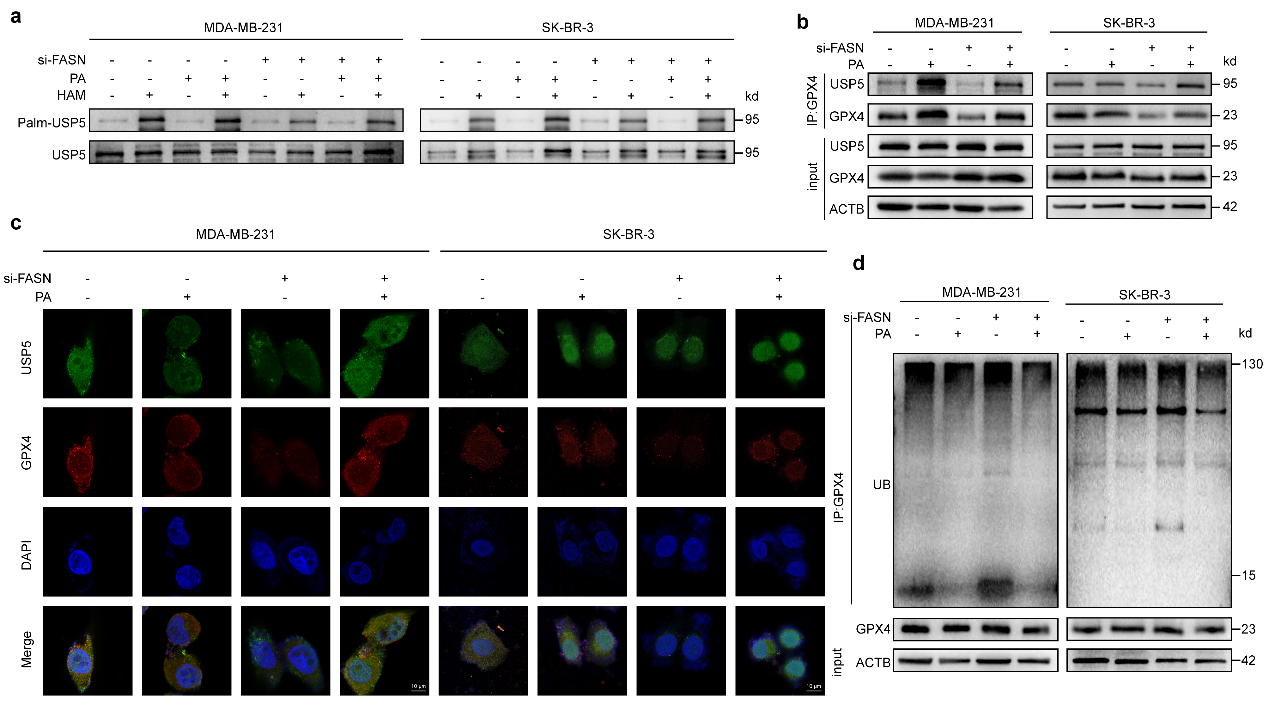


**Supplementary fig. 7** **Palmitic acid (PA)-induced regulation of USP5 palmitoylation and GPX4 stabilization.**

**a** Detection of USP5 palmitoylation via acyl-biotin exchange (ABE) assay in breast cancer cells treated with PA (0.4 Mm, 48 h) followed by MG132 (10 μM, 6 h). Immunoprecipitation (IP) was performed with anti-USP5 antibody, with hydroxylamine (HAM) treatment removing palmitoyl groups and free thiols labeled by BMCC-biotin (detected by streptavidin-HRP)**.** **b** GPX4 protein levels analyzed by IP after 48 h PA treatment. **c** Co-localization of USP5 (green) and GPX4 (red) in PA-treated cells by immunofluorescence (nuclei: DAPI, blue). **d** GPX4 ubiquitination assessment in PA-pretreated cells with MG132. Scale bars: 10 μm (**c**). Experiments were performed three times independently (**a**–**d**). P < 0.05 was considered to be statistically significant.


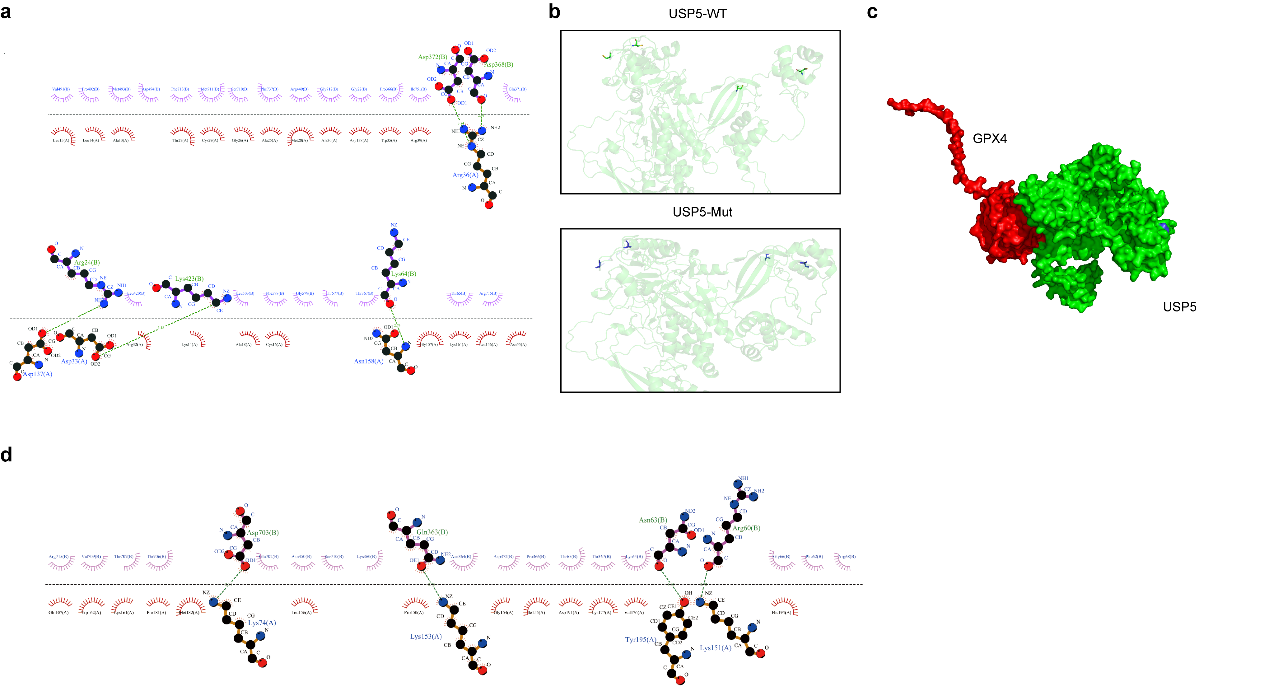


**Supplementary fig. 8** **Interactions and Structural Analysis of GPX4 and WT/Mutant USP5 Proteins.**

**a** Interacting residues at the GPX4 (Chain A, red lollipops)-USP5 (Chain B, purple lollipops) binding interface are shown. Hydrogen bonds and salt bridges are indicated by green and red dashed lines, respectively. **b** Structural schematic of USP5 mutations. **c** Docking model of GPX4 with mutant USP5. Shown in Suface + Cartoon representation. **d** 2D diagram of amino acid residues involved in the interface interaction between GPX4 protein and mutant USP5 protein.
