# Supplementary material for: FASN inhibits ferroptosis in breast cancer via USP5 palmitoylation-dependent regulation of GPX4 deubiquitination
Source: J Exp Clin Cancer Res. 2025 Oct 14;44:289. doi: 10.1186/s13046-025-03548-8 (PMC12523187; doi:10.1186/s13046-025-03548-8)
Supplement: Supplementary file 2 — Supplementary Material 2 [file 13046_2025_3548_MOESM2_ESM.docx]

**Supplementary Table 1** Scoring results of molecular docking between GPX4 protein and USP5 protein.

| Compound | Docking Score | Confidence score |
| --- | --- | --- |
| 1 | -204.72 |  |
| 2 | -203.52 |  |
| 3 | -201.51 |  |
| 4 | -201.35 |  |

**Supplementary Table 2** Intermolecular hydrogen bonding network between GPX4 and USP5 at their interaction interface.

| Residue index | Distance | Category | Type |
| --- | --- | --- | --- |
| A:Arg8:NE-B:Pro253:O | 2.63 | Hydrogen Bond | Conventional Hydrogen Bond |
| A:Arg5:NH1-B:Leu284:O | 2.19 | Hydrogen Bond | Conventional Hydrogen Bond |
| A:Leu24:O-B:Ser733:OG | 3.15 | Hydrogen Bond | Conventional Hydrogen Bond |
| A:Leu15:O-B:Gln167:NE2 | 2.58 | Hydrogen Bond | Conventional Hydrogen Bond |

Table Notes: A: Represents GPX4 protein. B: Represents USP5 protein.

**Supplementary Table 3** Docking score comparison of GPX4 interaction with wild-type versus mutant USP5.

| Before mutation | | After the mutation | |
| --- | --- | --- | --- |
| Docking_ Score | Confidence | Docking_ Score | Confidence |
| -211.68 | 0.774 | -192.28 | 0.699 |
| -201.95 | 0.739 | -190.96 | 0.694 |
| -196.93 | 0.719 | -188.65 | 0.684 |
| -193.73 | 0.704 | -187.93 | 0.681 |
| -192.03 | 0.699 | -185.31 | 0.670 |
| -191.65 | 0.697 | -181.80 | 0.654 |
| -190.01 | 0.690 | -180.38 | 0.647 |

**Supplementary Table 4** FASN and USP5 siRNA Sequences.

| Name | Sequence (5’ to 3’) |
| --- | --- |
| FASN siRNA-1 | ACAGACGAGAGCACCUUUGAUTT |
| FASN siRNA-2 | GGUAGUGAGUGGGAAGGUGUATT |
| FASN siRNA-3 | CCACAACAGCCUCUUCCUGUUTT |
| USP5 siRNA-1 | CCACAGAGAAGGUGAAGUATT |
| USP5 siRNA-2 | UCAACAUGGUGGAGAGGAATT |
| USP5 siRNA-3 | UGACUGAGUUGGAGAUAGATT |

**Supplementary Table 5** Primer Sequences for FASN, USP5, and GPX4.

| Primer Name | Sequence（5’ to 3’） |
| --- | --- |
| FASN forward | TGAGAGATGGCTTGCTGGA |
| FASN reverse | CCGCTGTACTTGGGCTTG |
| GPX4 forward | GAGGCAAGACCGAAGTAAACTAC |
| GPX4 reverse | CCGAACTGGTACACGGGAA |
| USP5 forward | GCTGCTGTCAGTATTACCGAC |
| USP5 reverse | AAAGCCCAGAAACGTGTTCATA |
